# Psychometric evaluation and community norms of the GAD-7, based on a representative German sample

**DOI:** 10.3389/fpsyg.2025.1526181

**Published:** 2025-03-20

**Authors:** Sören Kliem, Cedric Sachser, Anna Lohmann, Dirk Baier, Elmar Brähler, Jörg M. Fegert, Harald Gündel

**Affiliations:** ^1^Department of Social Welfare, Ernst-Abbe-Hochschule Jena - University of Applied Sciences, Jena, Germany; ^2^Department for Child and Adolescent Psychiatry/Psychotherapy, University Clinic for Psychosomatic Medicine and Psychotherapy Ulm, Ulm, Germany; ^3^Institute of Delinquency and Crime Prevention, Zurich University of Applied Sciences, Zurich, Switzerland; ^4^Department of Psychosomatic Medicine and Psychotherapy, University Medical Center of the Johannes Gutenberg University Mainz, Mainz, Germany; ^5^Department of Medical Psychology and Sociology, Leipzig University, Leipzig, Germany

**Keywords:** GAD-7, generalized anxiety, self-report questionnaire, population norms, psychometrics, measurement invariance

## Abstract

**Introduction:**

The Generalized Anxiety Disorder-7 (GAD-7) scale is widely used to assess generalized anxiety symptoms in both clinical and general populations. However, updated psychometric evaluations and population norms for the German adult population are lacking. This study aims to examine the psychometric properties of the GAD-7 and provide representative population norms.

**Methods:**

A representative sample of the adult population in Germany (N = 2,519) was assessed. Item characteristics (means, standard deviations, inter-item correlations) were examined. Construct validity was evaluated through correlations with the PHQ-9 and BSI-18. Internal consistency was assessed using coefficient omega. Confirmatory factor analysis (CFA) was conducted to test the one-factor model, and measurement invariance across gender and age was examined using multi-group CFA.

**Results:**

The GAD-7 demonstrated strong internal consistency and construct validity. CFA supported the assumed one-factor model. Measurement invariance analyses indicated that the GAD-7 provides comparable measurements across gender and age groups. Updated population-based norms were established for the total sample and specific age groups.

**Discussion:**

Findings confirm the GAD-7 as a psychometrically sound measure for generalized anxiety in the general population. The updated norms enhance its applicability in clinical and epidemiological research, supporting its use for screening and assessment across diverse demographic groups.

## 1 Introduction

Generalized anxiety disorder (GAD) is among the most prevalent mental disorders (Remes et al., 2016; Somers et al., 2006), yet it is generally thought to be underdiagnosed (Parmentier et al., 2013) and undertreated (Ruscio et al., 2017). Brief and effective screening tools are essential for identifying and assessing anxiety symptoms in both clinical and research settings. The Generalized Anxiety Disorder-7 (GAD-7) scale is widely used for this purpose, having been validated against diagnostic clinical interviews to establish its sensitivity, specificity, and predictive values (Spitzer et al., 2006). Originally developed for primary care settings, the GAD-7 has also been employed in epidemiological studies (e.g., Hinz et al., 2017; Johansson et al., 2013; Löwe et al., 2008) and has seen extensive use in public health monitoring, including the National Health Interview Survey (NHIS) (Terlizzi and Zablotsky, 2024) and COVID-19 research (Fancourt et al., 2021; McBride et al., 2021).

The GAD-7 has been translated into numerous languages, including Spanish, French, German, Chinese, Japanese, Korean, Arabic, Portuguese, Russian, and Turkish and validated across diverse patient populations (see Plummer et al., 2016, for an overview). However, despite its widespread use, research on normative scores remains limited (Hinz et al., 2017). Normative values are crucial for contextualizing individual and group distress levels. To date, only one large-scale normative study has been conducted in a general population sample in Germany (Löwe et al., 2008). Other general population studies confirm strong psychometric properties, including high internal consistency and factorial validity, supporting the one-dimensional structure of the scale (e.g., South Korea: Ahn et al., 2019), (Belgium: De Man et al., 2021), (Spain: Garcia-Campayo et al., 2010), (Brazil: Monteiro et al., 2022). Shevlin et al. (2020) established measurement invariance of the GAD-7 across four European countries (UK, Ireland, Spain, and Italy), further supporting its cross-cultural validity. A recent large-scale Japanese study provided valuable prevalence data but did not report psychometric properties or norms (Matsuyama et al., 2024). Moreover, the GAD-7 has demonstrated robust performance in web-based assessments (Donker et al., 2011).

Previous studies examining the psychometric properties of the GAD-7 in the German general population have primarily focused on its factorial structure, internal consistency, and criterion validity. Löwe et al. (2008) originally validated the German version, demonstrating strong internal consistency (Cronbach's α>0.85) and high correlations with related constructs such as depression (PHQ-2, r = 0.64, p < 0.001) and self-esteem (Rosenberg Self-Esteem Scale, r = −0.43, *p* < 0.001). They found CFA support for an one-factor model with high factor loadings ranging from 0.76 to 0.90. Hinz et al. (2017) reports the same high internal consistency and also confirmed the unidimensional structure of the scale via confirmatory factor analysis (CFA). They furthermore supported the scale's measurement invariance across age and gender. Both studies found consistently higher anxiety scores in women.

Population-level shifts in anxiety symptoms underscore the need for regularly updated normative data. Establishing community norms for the GAD-7 is essential for contextualizing individual and group scores, facilitating interpretation relative to the general population. Norms provide a reference framework to distinguish typical from elevated anxiety levels, aiding in both clinical and epidemiological applications (Spitzer et al., 2006; Löwe et al., 2008). They also enable comparisons across demographic groups, contributing to a more nuanced understanding of anxiety prevalence and disparities (Hinz et al., 2017) Recent findings by Hinz et al. (2023) confirmed longitudinal measurement invariance over six years in Germany and identified a statistically significant increase in anxiety symptoms, emphasizing the necessity for contemporary reference values.

The present study evaluates the psychometric properties of the GAD-7 in a large, representative German community sample and provides updated norms. We test evidence based on internal structure by assessing its reliability and unidimensional factor structure and hypothesize that the GAD-7 demonstrates high internal consistency and configural, metric, and scalar invariance across gender and age. To establish evidence based on relations to other variables, we hypothesize strong positive associations with depressive symptoms and somatization and furthermore expect differences in anxiety levels across gender. Evidence based on test content and response processes is not the focus of this study, as the GAD-7 is a well-established measure with extensive prior validation. By addressing these aspects, this study provides a comprehensive update on the scale's psychometric properties and its interpretability in the general population.

### 1.1 Procedure

The GAD-7 was presented as part of a large survey conducted by Leipzig University between December 2020 and March 2021. The survey was carried out by the contractor USUMA Markt- und Sozialforschung an independent institute for opinion and social research. The goals of the survey were (a) to assess prevalence rates of a variety of relevant physical or mental disorders and related risk behaviors (descriptive epidemiology), (b) to examine causes and conditions of these disorders (analytic epidemiology), and (c) to analyze psychometric properties and provide German population norms for clinical-psychological instruments. The survey consisted of two parts. The first part was interviewer guided and consisted of demographic as well as household information in accordance with the principles of the German Statistisches Bundesamt (Federal Statistical Office). In the second part the participants filled in paper-based questionnaires themselves in the presence of but independent as well as out of sight of the interviewer. The interviewer was, however, available for questions. Informed consent was obtained from all participants prior to the interview. Minimum age for participation was 16 years. For under-aged participants at least one legal guardian was informed about the sampling procedure and the contents of the survey. All participants were provided a written copy of the confidentiality agreement containing details regarding the handling of their personal information. The study was conducted in accordance with the Declaration of Helsinki. All procedures were approved by the Ethics Committee of the Medical Faculty of the University of Leipzig (Az.: 474/20-ek).

### 1.2 Sample description

**Figure 1:**
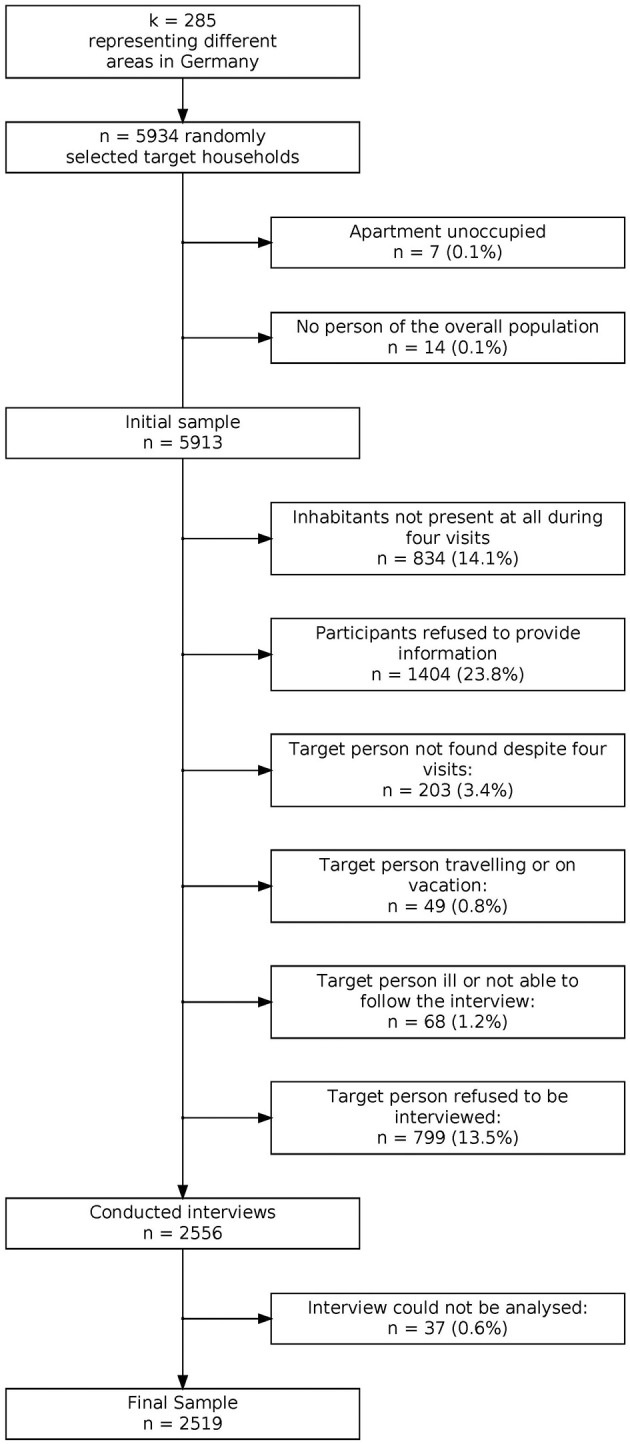
Flowchart of sampling procedure and reasons for nonparticipation. Reproduced from Kliem et al. (2024).

The sampling was conducted according to the ADM sampling system F2F. This procedure consists of three steps. In a first step, the area of the federal republic of Germany is divided into regions of which 258 are sampled with sampling probability proportional to the number of households. In a second step, 5,676 households are selected based on a random route procedure. The target person within each household is identified using a Kish selection grid. Details regarding response can be obtained from Figure 1. The following analyses are based on data from *N* = 2,519 participants which corresponds to a response rate of 42 %. Figure 1 presents a detailed overview of the sampling procedure and reasons for non-response. Sample descriptives can be found in Table 1.

**Table 1:** Demographic characteristics of the study sample.

	**Male**	**Female**	**Diverse**	**Total**
	**(*****N*** = **1,193)**	**(*****N*** = **1,322)**	**(*****N*** = **4)**	**(*****N*** = **2,519)**
**Age (years)**				
Mean (SD)	50.1 (17.7)	50.5 (18.3)	44.8 (26.5)	50.3 (18.1)
Median [Min, Max]	52.0 [16.0, 96.0]	51.0 [16.0, 96.0]	41.5 [21.0, 75.0]	51.0 [16.0, 96.0]
**Age categories**				
16–24	102 (8.5%)	125 (9.5%)	2 (50.0%)	229 (9.1%)
25–34	190 (15.9%)	174 (13.2%)	0 (0%)	364 (14.5%)
35–44	174 (14.6%)	225 (17.0%)	0 (0%)	399 (15.8%)
45–54	195 (16.3%)	216 (16.3%)	0 (0%)	411 (16.3%)
55–64	244 (20.5%)	243 (18.4%)	1 (25.0%)	488 (19.4%)
65–74	190 (15.9%)	200 (15.1%)	0 (0%)	390 (15.5%)
75+	98 (8.2%)	139 (10.5%)	1 (25.0%)	238 (9.4%)
**Nationality**				
German	1,151 (96.5%)	1,271 (96.1%)	3 (75.0%)	2,425 (96.3%)
Not German	42 (3.5%)	48 (3.6%)	1 (25.0%)	91 (3.6%)
Missing	0 (0%)	3 (0.2%)	0 (0%)	3 (0.1%)
**Marital status**				
Married/living together	547 (45.9%)	527 (39.9%)	2 (50.0%)	1,076 (42.7%)
Married/separated	40 (3.4%)	25 (1.9%)	0 (0%)	65 (2.6%)
Single	398 (33.4%)	357 (27.0%)	2 (50.0%)	757 (30.1%)
Divorced	143 (12.0%)	227 (17.2%)	0 (0%)	370 (14.7%)
Widowed	62 (5.2%)	181 (13.7%)	0 (0%)	243 (9.6%)
Missing	3 (0.3%)	5 (0.4%)	0 (0%)	8 (0.3%)
**Living with partner**				
Living with partner	729 (61.1%)	737 (55.7%)	2 (50.0%)	1,468 (58.3%)
Not living with partner	444 (37.2%)	565 (42.7%)	2 (50.0%)	1,011 (40.1%)
Missing	20 (1.7%)	20 (1.5%)	0 (0%)	40 (1.6%)

### 1.3 Instruments

As the survey served multiple epidemiological purposes, only those measures that were used in the validation process are discussed in this paper. In addition to extensive demographic information (see Table 1), health related behavior, such as the number of sick days, doctor visits, and hospital stays, were assessed. The following measures were used for the validation of the scales at hand.

#### 1.3.1 The general anxiety disorder scale (GAD-7)

The GAD-7 (Spitzer et al., 2006) is a brief self-report scale with seven items assessing generalized anxiety. Each of the seven items is rated on a scale from 0 (not at all) to 3 (almost every day). The total score of the GAD-7 scale ranges from 0 to 21. When formally validated using diagnostic psychiatric interviews, a GAD-7 score of ≥ 10 has been found to have a sensitivity of 89% and a specificity of 82% for detecting generalized anxiety disorder (Spitzer et al., 2006). The GAD-7 showed high internal consistency in previous general population studies (α = 0.89; Löwe et al., 2008).

#### 1.3.2 Patient health questionnaire (PHQ-9)

The PHQ-9 (Kroenke et al., 2001) is a self-report scale, that scores depression symptoms on a four-point scale from 0 (not at all) to 3 (almost every day), providing a total severity score ranging from 0 to 27. In the present study, the German version of the PHQ-9 (Martin et al., 2006) was used. To assess internal consistency within our sample, we computed Cronbach's alpha and McDonald's omega. The PHQ-9 demonstrated high internal consistency in the study at hand (α = 0.90, 95% CI [0.89, 0.91]; ω = 0.93, 95% CI [0.92, 0.94]), which aligns with findings from previous general population studies (α = 0.87; Kocalevent et al., 2013).

#### 1.3.3 The brief symptom inventory (BSI-18)

The BSI-18 (Derogatis and Fitzpatrick, 2004) is an 18-item short form of the Symptom-Checklist 90-R. It contains three subscales of six items each: somatization (SOMA), depression (DEPR), and anxiety (ANX). The sum score of all 18 items can furthermore be interpreted as a Global Severity Index (GSI). To evaluate internal consistency in our sample, we calculated Cronbach's alpha and McDonald's omega. The BSI-18 showed high reliability in the study at hand (α = 0.93, 95% CI [0.92, 0.94]; ω = 0.93, 95% CI [0.93, 0.94]), consistent with previous general population studies [α = 0.93 (GSI), 0.82 (SOMA), 0.87 (DEPR), 0.84 (ANX); Franke et al., 2017].

### 1.4 Statistical analysis

#### 1.4.1 Missing data

To account for missing data, we applied chained equation modeling (see van Buuren and Groothuis-Oudshoorn, 2011) using the following variables: gender, age, nationality, marital status, living with a partner as well as all items from the scales GAD-7, PHQ-9 and BSI-18 to estimate missing data (proportion of missing data of the GAD-7 items: 0.30%–0.70%). To avoid implausible item values, the estimated values (ŷ) were corrected by predictive mean matching (i.e., the observable values closest to the predicted value were chosen). We used the R package mice (van Buuren and Groothuis-Oudshoorn, 2011) for imputation.

#### 1.4.2 Item characteristics

Mean and standard deviations were obtained for all GAD-7 items in the total sample as well as for the sub samples of male and female gender. Effect sizes of group differences in item means were computed as Cohen's d and Cliff's δ. Item correlations were obtained. To assess item characteristics we examined the skewness and kurtosis, item difficulty (i.e. percentage of participants endorsing each item), item-total correlations, and Cronbach's alpha if the item was deleted. Item characteristics were obtained unsing the R package psych (William Revelle, 2024).

#### 1.4.3 Construct validity

To assess construct validity of the GAD-7, we correlated the scale with the PHQ-9 and the three BSI-18 subscales (somatization, anxiety and depression) as well as with the BSI global severity index. The following hypotheses were formulated: anxiety levels should be higher in individuals with (a) higher depression scores (e.g. Byrd-Bredbenner et al., 2021; Hinz et al., 2017; Löwe et al., 2008), and (b) higher somatization scores (e.g. Gierk et al., 2014; Kliem et al., 2017a).

### 1.5 Population norms

Population norms are computed as cumulative percentiles. Female and male subsamples as well as several of age groups are tabulated separately. To avoid spurious jumps we also provide smoothed norms, which were obtained via a shape-constrained additive modeling (SCAM) approach. SCAM utilizes penalized regression splines with built-in monotonicity constraints, ensuring that the resulting curves are non-decreasing–a critical property for cumulative distributions. The smoothing was implemented using the scam package in R (Pya, 2023).

#### 1.5.1 Internal consistency

Considering that coefficient α could be affected by problems stemming from its assumptions not being met (McNeish, 2018), internal consistency of the GAD-7 is reported additionally as McDonald's ω which is computed using the R package semTools (Jorgensen et al., 2021). To evaluate the internal consistency of the GAD-7 scale across severity categories, McDonald's omega was computed using polychoric correlations and the psych package (William Revelle, 2024). This approach was chosen due to the challenges in obtaining reliable CFA-based omega estimates in subgroup analyses.

#### 1.5.2 Factorial validity and measurement invariance

To test the one-dimensional structure of the GAD-7, confirmatory factor analyses (CFA) were conducted. CFAs were performed using the lavaan package (Rosseel, 2012) for R statistics. As suggested for the use with ordered categorical measures, weighted least square means and variance adjusted estimation (WLSMV) was used. Measurement invariance (MI) was tested using multiple group factor analysis (MGCFA). Following the procedure suggested by Wu and Estabrook (2016) for ordered categorical variables, we used theta parameterization and identified the model by setting the means and variances of the latent factors to 0 and 1, respectively, item intercepts to 0 and residual variances to 1. The following models were subsequently tested: configural invariance (no constraints apart from those necessary for model identification), threshold invariance (constraining all thresholds to be equal), weak invariance (constraint of loadings), strong invariance (constraining of intercepts), and full invariance (constraining residual invariance). See Supplementary Figure A1 for a path model overview of the structural equation models assessed. Chen (2007) suggests the following cut-off criteria: a change of < -0.01 in CFI in addition to a change of ≥ 0.015 in RMSEA indicating non-invariance. We conducted MGCFAs for the GAD-7 one-factor model across gender, age (below median age vs. above median age), as well as age x gender. Due to the low number of individuals classifying as neither male nor female, these cases were not included in the MGCFA's of gender and age x gender. MI analyses were conducted using the semTools package (Jorgensen et al., 2021) for R statistics.

#### 1.5.3 Sensitivity analysis—Careless response patterns

To evaluate the robustness of the results, a sensitivity analysis was conducted by excluding respondents with inconsistent response patterns. Outliers were identified based on G+ scores, a measure of Guttman errors, which occur when respondents endorse harder items but not easier ones, violating the expected hierarchical order of items (Van der Ark, 2012). The G+ scores were calculated using the check.errors() function from the mokken package in R (Van der Ark, 2012). Outliers were defined using Tukey's method, with scores exceeding the upper fence (Q3+3 × IQR) flagged as discordant (Tukey, 1977). A total of 87 outliers (3.5% of the sample) were excluded, as inconsistent response patterns can bias psychometric results and distort conclusions. Analyses were repeated without these cases to assess the sensitivity of the findings.

## 2 Results

### 2.1 Item characteristics

Supplementary Table A3 displays means and standard deviations for the seven items of the GAD-7 in the total sample as well as effect sizes for mean differences regarding gender. On the item-level there was a consistent pattern of female participants exhibiting higher mean scores as well as higher variability on most GAD-7 items. Effect sizes (Cohen's d) of these mean differences ranged from d = -0.02 [-0.1,0.05] to d = -0.16 [-0.24,-0.08]. Item characteristics can be obtained from Supplementary Table A6. Supplementary Table A7 presents GAD-7 item-level associations with PHQ-9 and BSI-18 subscales. Supplementary Figure A3 displays the distribution of responses across the seven GAD-7 items.

### 2.2 Construct validity

To determine evidence of construct validity of the GAD-7 correlation coefficients were calculated with related instruments. In line with our hypotheses, there were high positive correlations between the GAD-7 and measures of somatization, anxiety and depression as assessed by BSI-18 sub scales (see Supplementary Table A5). In the same vein, the PHQ-9 assessing depression showed positive correlations with the GAD-7.

Supplementary Table A7 presents the associations between individual GAD-7 items and the PHQ-9, as well as the Global Severity Index, Somatization, Anxiety, and Depression subscales of the BSI-18, using both the Maximum Information Coefficient (MIC) and Spearman's rank correlation (ρ_*s*_). As expected, all items show positive associations with these scales, with the strongest relationships observed with the PHQ-9 rather than the anxiety subscale of the BSI-18. This pattern may reflect the substantial overlap between generalized anxiety and depressive symptoms. Additionally, the stronger association with the PHQ-9 may be partly attributable to the identical response scaling of both measures.

### 2.3 Population norms

Prevalences according to the severity cut-offs suggested by Spitzer et al. (2006) can be found in Table 2.

**Table 2:** Percentage of participants per severity level based on Spitzer et al. (2006) GAD-7 cut-offs.

**GAD-7 severity**	**Total, %**	**Male, %**	**Female, %**
Minimal (0–4)	81.30	83.40	79.50
Mild (5–9)	14.37	12.82	15.73
Moderate (10–14)	3.29	2.77	3.78
Severe (15–21)	1.03	1.01	0.98

Table 3 shows cumulative percentiles of GAD-7 scores for the total sample. Additional norms split by gender as well as age group can be found in the Supplementary Tables A9, A11. Smoothed norms are also available in the Supplementary Tables A8, A10, A12.

**Table 3:** Population based norms (cumulative percentiles) of the GAD-7 scores (total sample).

**GAD-7**	**Total**	**Age 16–24**	**Age 25–34**	**Age 35–44**	**Age 45–54**	**Age 55–64**	**Age 65–74**	**Age 75+**
0	47.8	53.3	54.9	51.9	43.3	43.0	47.7	42.0
1	60.5	61.6	68.7	63.4	60.1	54.7	61.0	54.2
2	70.1	65.5	75.0	71.7	69.6	66.6	72.3	68.5
3	76.7	71.2	79.1	78.4	76.2	77.0	77.4	73.9
4	81.3	76.0	84.6	83.2	81.3	80.5	82.1	78.6
5	85.9	84.7	87.4	87.0	86.1	84.6	86.9	84.0
6	89.4	88.2	91.8	90.0	90.5	86.9	90.5	87.8
7	93.2	93.0	95.3	92.0	94.6	92.4	94.1	89.9
8	94.7	93.9	97.5	92.7	96.1	94.1	95.4	92.0
9	95.7	95.6	98.1	93.7	97.1	95.3	95.6	93.7
10	96.7	96.5	98.1	95.0	98.3	96.3	96.7	95.4
11	97.5	96.9	99.2	95.5	98.5	97.1	97.7	97.9
12	98.0	96.9	99.5	96.2	98.8	97.1	98.2	99.6
13	98.5	98.7	99.7	97.0	99.0	97.5	98.7	>99.9
14	99.0	99.1	>99.9	97.2	99.8	98.2	99.2	>99.9
15	99.2	99.6	>99.9	98.2	99.8	98.2	99.5	>99.9
16	99.4	>99.9	>99.9	98.5	>99.9	98.4	99.7	>99.9
17	99.6	>99.9	>99.9	99.0	>99.9	98.6	>99.9	>99.9
18	99.7	>99.9	>99.9	99.2	>99.9	99.2	>99.9	>99.9
19	99.8	>99.9	>99.9	99.5	>99.9	99.4	>99.9	>99.9
20	99.8	>99.9	>99.9	99.7	>99.9	99.4	>99.9	>99.9
21	>99.9	>99.9	>99.9	>99.9	>99.9	>99.9	>99.9	>99.9

### 2.4 Internal consistency

Cronbach's alpha of the GAD-7 for the full sample was α = 0.9, 95% CI (0.89, 0.91). McDonald's omega of the GAD-7 for the full sample was ω = 0.92, 95% CI (0.91, 0.93).

The reliability estimates suggest that the GAD-7 exhibits strong internal consistency in the minimal (ω = 0.78) and severe (ω = 0.72) categories. However, the reliability is notably lower in the mild (ω = 0.53) and moderate (ω = 0.65) categories. This pattern may reflect differences in item functioning or response variability across severity groups.

In the minimal and severe groups, participants may exhibit more uniform response patterns, either due to consistently low or consistently high anxiety levels, leading to higher internal consistency. In contrast, the mild and moderate groups might display greater variability in responses, potentially reflecting more heterogeneity in the manifestation of anxiety symptoms within these groups.

### 2.5 Factorial validity

A CFA was conducted to assess the unidimensional structure of the GAD-7. The fit indices indicated reasonable model fit, with a robust CFI of 0.936, a robust TLI of 0.905, and an SRMR of 0.033. However, the robust RMSEA was 0.176 (90% CI [0.157, 0.196]), suggesting some misfit.

To improve the model, we inspected modification indices and introduced residual correlations between items with overlapping content: #2 (Not able to stop/control worrying) with #3 (Worrying too much). These adjustments improved the fit indices (CFI = 0.961, RMSEA = 0.143).

Despite these modifications, strong standardized factor loadings (0.82–0.91, a path diagram can be found in Supplementary Figure A2) of the speak in favor of a one factor solution.

### 2.6 Measurement invariance

The fit measures obtained in the measurement invariance analyses of the GAD-7 are presented in Supplementary Table A2. Adequate fit was observed across all levels of invariance testing, with CFI values consistently ≥0.998 and RMSEA values decreasing as model constraints were added. For gender, configural invariance showed a good fit (CFI = 0.998, RMSEA = 0.072), and full invariance was supported (CFI = 0.998, RMSEA = 0.045) with negligible changes in model fit (ΔCFI = 0, ΔRMSEA ≤ −0.009). Similarly, for age groups, configural invariance yielded CFI = 0.998 and RMSEA = 0.077, with full invariance confirmed (CFI = 0.998, RMSEA = 0.051, ΔCFI = 0, ΔRMSEA ≤ −0.01). When testing invariance across both gender and age simultaneously, model fit remained stable (configural: CFI = 0.999, RMSEA = 0.074; full invariance: CFI = 0.998, RMSEA = 0.047, ΔCFI = 0, ΔRMSEA ≤ −0.013).

## 3 Discussion

The present study investigates the psychometric quality of the GAD-7 using a large and representative sample of the German general population. Based on coefficient ω, the GAD-7 demonstrates high internal consistency, reinforcing its reliability as a screening tool. The lower reliability observed in the mild and moderate severity categories may indicate greater heterogeneity in symptom manifestation within these groups. This aligns with prior research suggesting that individuals with moderate anxiety levels may experience a broader range of symptoms or more nuanced symptom profiles (e.g., Terlizzi and Zablotsky, 2024). In contrast, participants in the minimal and severe groups likely exhibit more consistent response patterns, reflecting either an absence or a pronounced intensity of symptoms. These findings highlight the importance of further examining item functioning and variability across the severity spectrum to ensure the robustness of the GAD-7 across diverse populations.

Furthermore, measurement invariance testing using MGCFA confirmed comparable factor structures across gender and age groups. The model fit statistics indicated that the assumed factor structure of the GAD-7 holds across these subgroups, supporting its utility for comparative analyses across demographic variables. This provides validity evidence based on internal structure, following the framework outlined in the “Standards” (American Educational Research Association et al., 2014). Additionally, the observed correlations between the GAD-7 and other established measures of psychological distress (PHQ-9, BSI-18) align with prior research, further supporting validity evidence based on relationships with other variables (Löwe et al., 2008). Overall, the results affirm that the GAD-7 is an efficient, reliable, and valid instrument for assessing generalized anxiety in the German population.

A key contribution of this study is the provision of updated norm tables, the primary aim of this research. These percentiles (see Table 3, Supplementary Tables A8–A11) are stratified by age and available in both gender-specific and gender-neutral formats. A clinical cut-off of 10 points has been suggested in previous research (Spitzer et al., 2006), which, in our sample, corresponds to the 96th-99th percentiles. This indicates symptom severity well above the population mean, ranging from 1.75 to 2.33 standard deviations above the mean. It is important to note that these normative data are provided as reference values for clinical interpretation and are not intended to serve as definitive clinical norms. Compared to the norms presented by Hinz et al. (2017), which were derived from a sample in Leipzig (a large city in eastern Germany, population: 600,000), the present study benefits from a sample representative of the entire Federal Republic of Germany. Substantially lower item means were observed across all items except for item 7 (“Feeling afraid”), where endorsement rates were similar. Given socio-structural differences between eastern and western Germany, as well as between urban and rural regions, it is conceivable that the previous norms (Hinz et al., 2017) do not generalize to the entire German population.

The percentiles obtained in this study deviate considerably from prior normative studies (Erhardt et al., 2022; Hinz et al., 2017; Löwe et al., 2008). A notably high proportion of participants reported minimal generalized anxiety symptoms. This decline in anxiety levels aligns with recent population-level data from Erhardt et al. (2022), based on data from 2014-2019, which reported the following prevalence rates: minimal (0-4): 74.9.

Several large surveys assessing the GAD-7 have been conducted in Germany (Bäuerle et al., 2020; Skoda et al., 2021; Streit et al., 2022) and other European countries (Hyland et al., 2021; José et al., 2023). While GAD symptom burden varied across these studies, all reported substantially higher anxiety levels than the present study. Notably, these studies employed large online convenience samples and framed their surveys around pandemic-related mental health concerns. Given the potential for self-selection bias in such studies, the present results, derived from a representative face-to-face survey, provide an important counterbalance to prevailing narratives.

Mean GAD-7 scores in this study (M = 2.18, SD = 3.28) were substantially lower than those observed in recent nationally representative samples from other countries [e.g., UK: 5.25 (5.68), Ireland: 5.03 (5.52), Spain: 5.86 (5.24), Italy: 5.73 (5.14)] (Shevlin et al., 2022). This aligns with global epidemiological data from Ruscio et al. (2017), which identified Germany as having a relatively low prevalence of generalized anxiety disorder compared to other nations. Taken together with findings on internal consistency, these results underscore the need for careful monitoring of item wording to ensure that the GAD-7 captures the nuances of anxiety symptoms across different severity levels.

These findings also emphasize the necessity of regularly updating norms and caution against assuming stable prevalence rates over time. For example, Gottschick et al. (2023) found elevated anxiety symptoms in the German population during the onset of the Russo-Ukrainian war compared to the COVID-19 pandemic period. This underscores the importance of continuously revising normative data to ensure clinical relevance. Our study provides a critical reference point for longitudinal research, offering insights into symptom variability at the population level.

### 3.1 Practical implications

The findings of this study hold significant practical implications for both clinical and research settings. First, the provision of updated norm tables allows for improved screening and diagnostic decision-making by healthcare professionals. By offering percentile-based reference values, clinicians can better interpret individual GAD-7 scores relative to the broader population, aiding in the identification of individuals at risk for generalized anxiety disorder. We emphasize that while these norms serve as valuable reference data in clinical contexts, they are derived from a representative general population sample and should not be used as definitive clinical norms. Second, the confirmation of measurement invariance across gender and age groups ensures that the GAD-7 can be reliably used in diverse demographic settings, facilitating comparisons across subpopulations. Third, given the observed decline in anxiety symptoms and divergence from prior norms, this study highlights the importance of considering contemporary population trends when applying psychological assessments. Researchers and practitioners should remain cautious about relying on outdated reference values, particularly when tracking prevalence changes over time. Finally, these results contribute to the broader discourse on mental health epidemiology by emphasizing the role of socio-structural factors in shaping anxiety prevalence. Future research should continue to examine how economic, political, and public health events influence mental health trajectories at the population level.

### 3.2 Limitations

Despite the many strengths of this study (especially the representativeness of the sample), certain limitations must be mentioned. The response rate is only 42.6%. However, lower response rates than in clinical studies are quite common in general population studies, and the response rate of this study was comparable to similar surveys (e.g. Kliem et al., 2015, 2018, 2017b). Although considerable efforts have been made to maximize the representativeness of the sample, a certain level of non-response is inevitable within the current design, raising concerns about potential bias stemming from this non-response. Unfortunately, a systematic evaluation of non-response bias is not feasible due to the lack of demographic information on those who did not respond. Such an assessment would require access to registry data, which is not readily available in Germany without government authorization. Furthermore, the diagnostic efficiency of the GAD-7 could not be examined because no additional clinical interviews were conducted.

### 3.3 Conclusion

In summary, the German GAD-7 has proven to be reliable and valid instruments for the use in different frameworks. Based on the (potentially COVID-19 related) change in symptom burden which the norm values reported in this study indicate we suggest to update the norms again in the near future to see whether the pandemic has to be interpreted as an interlude or “the new normal”.

## Data Availability

The datasets presented in this article are not readily available because ethics review board approval did not includes data sharing. Requests to access the datasets should be directed to Harald Gündel, harald.guendel@uniklinik-ulm.de.
