# Supplementary material for: Psychometric evaluation and community norms of the GAD-7, based on a representative German sample
Source: Front Psychol. 2025 Mar 20;16:1526181. doi: 10.3389/fpsyg.2025.1526181 (PMC11967371; doi:10.3389/fpsyg.2025.1526181)
Supplement: Supplementary file 1 [file Data_Sheet_1.pdf]

Supplementary materials

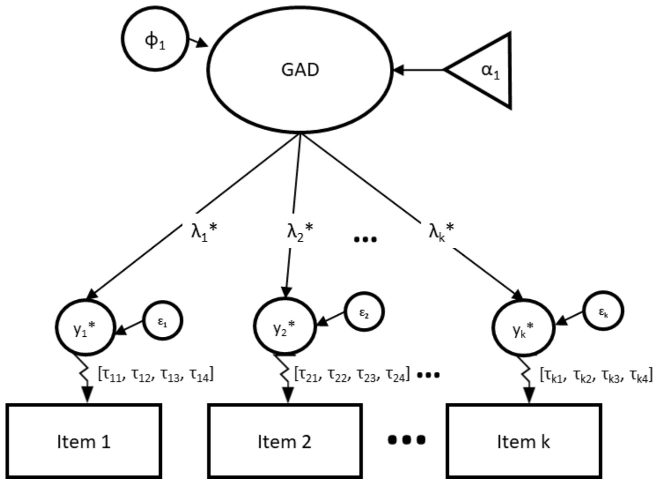

Figure A1 . MGCFA models for Measument Invariance Analysis:

(1) Configural invariance: Same CFA model fit in all groups;

(2) Threshold Invariance: thresholds $\tau_{1i}...\tau_{4i}$ constrained to be equal across groups;

(3) Metric Invariance: additionally, factor loadings λ_i constrained to be equal across groups;

(4) Scalar invariance: additionally, intercepts constrained to be equal across groups;

(5) Residual invariance: additionally, residual variances ϵ_i constrained to be equal across groups

Table A1. Parameter Constraints for MGCFA

Parameters	Threshold invariance `group.equal = "thresholds"*`	Metric Invariance group.equal = `c("thresholds, "loadings")*`	Scalar Invariance group.equal = `c("theresholds", "loadings", "intercepts")*`	Residual Invariance group.equal = `c("thresholds", "loadings", "intercepts")*`
Item loadings	free	equal across groups	equal across groups	equal across groups
Intercepts				
Items	constrained to 0 in first group	constrained to 0 in first group	equal (0) across groups	equal (0) across groups
Latent	constrained to 0 in all groups	constrained to 0 in all groups	constrained to zero in first group	constrained to zero in first group
Thresholds	equal across groups	equal across groups	equal across groups	equal across groups
Variances				
Residual	constrained to 1 in first group	constrained to 1 in first group	constrained to 1 in first group	equal (1) across groups
Latent	constrained to 1 in all groups	constrained to 1 in first group	constrained to 1 in first group	constrained to 1 in first group

Note: *setting for call of the `measEq.syntax()` function from the `semTools` package

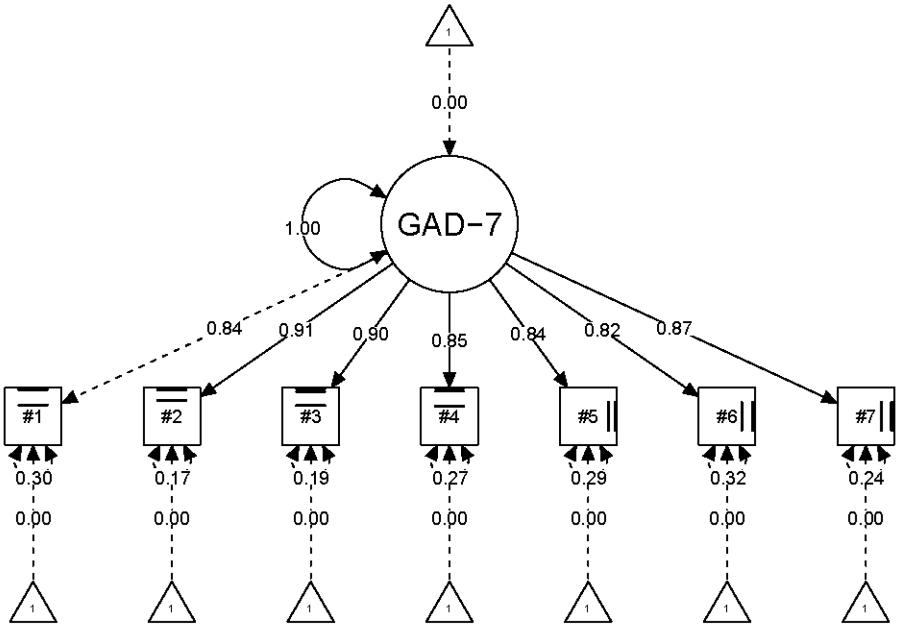

FigureA2. One factor CFA model of the GAD-7.

Table A2

Results of measurement invariance analyses

	χ^2	df	CFI	ΔCFI	RMSEA	ΔRMSEA	Measurement invariance
Gender (male, female)							
Configural invariance	99.259	28	0.998	-	0.072	-	-
Threshold invariance [1]	101.502	35	0.999	0	0.063	-0.009	✓
Metric invariance [2]	105.499	41	0.999	0	0.055	-0.008	✓
Scalar invariance [3]	121.643	47	0.998	0	0.052	-0.003	✓
Full invariance [4]	128.953	54	0.998	0	0.045	-0.007	✓
Age (<= 51, >51)							
Configural invariance	111.469	28	0.998	-	0.077	-	-
Threshold invariance [1]	118.461	35	0.998	0	0.069	-0.008	✓
Metric invariance [2]	119.819	41	0.998	0	0.059	-0.01	✓
Scalar invariance [3]	148.052	47	0.998	0	0.058	-0.001	✓
Full invariance [4]	156.073	54	0.998	0	0.051	-0.007	✓
Age x Gender							
Configural invariance	124.284	56	0.999	-	0.074	-	-
Threshold invariance [1]	134.041	77	0.999	0	0.061	-0.013	✓
Metric invariance [2]	144.856	95	0.999	0	0.051	-0.01	✓

Table A2 continued

	χ^2	df	CFI	ΔCFI	RMSEA	ΔRMSEA	Measurement invariance
Scalar invariance [3]	203.585	113	0.998	-0.001	0.053	0.003	✓
Full invariance [4]	233.188	134	0.998	0	0.047	-0.006	✓

Note. All fit statistics are robust; CFI = Comparative Fit Index; CFI = CFI-differences for the different measurement invariance levels; RMSEA = Root Mean Square Error of Approximation; RMSEA = RMSEA- differences for the different measurement invariance levels; ✓= ΔCFI < -.010 complemented by RMSEA ≥ .015 indicates a violation of measurement invariance; marks measurement invariance for the respective level; [1] equivalency of thresholds; [2] equivalency of thresholds + factor loadings; [3] equivalency of thresholds + factor loadings + equivalency of constants; [4] equivalency of thresholds + factor loadings + equivalency of constants + unique-factor variances.

Table A3

Means (M), standard deviation (SD), and group differences for the GAD-7 items

	Total		Male		Female		Group differences				
	M	SD	M	SD	M	SD	d [95% CI]	δ_{Cliff}	t	df	p
GAD-7	2.18	3.28	1.97	3.21	2.35	3.32	-0.11 [-0.2, -0.03]	-0.08	-2.86	2513	0.004
#1 Nervous/ anxious/ on edge	0.34	0.57	0.31	0.54	0.38	0.59	-0.13 [-0.2, -0.05]	-0.06	-3.15	2513	0.002
#2 Not able to stop/control worrying	0.27	0.56	0.24	0.55	0.29	0.57	-0.08 [-0.16, 0]	-0.04	-1.98	2513	0.048
#3 Worrying too much	0.40	0.67	0.36	0.65	0.44	0.68	-0.11 [-0.2, -0.04]	-0.06	-2.85	2513	0.004
#4 Trouble relaxing	0.39	0.64	0.35	0.62	0.42	0.64	-0.1 [-0.17, -0.02]	-0.05	-2.47	2513	0.014
#5 Restless	0.18	0.49	0.18	0.49	0.19	0.50	-0.03 [-0.1, 0.06]	-0.01	-0.66	2513	0.506
#6 Easily annoyed/irritable	0.30	0.59	0.30	0.60	0.31	0.58	-0.02 [-0.1, 0.05]	-0.02	-0.48	2513	0.632
#7 Feeling afraid	0.29	0.59	0.24	0.55	0.33	0.63	-0.16 [-0.24, -0.08]	-0.07	-4.02	2513	<0.001

Note. GAD = Generalized Anxiety Disorder Assessment, d = Cohen's d with bootstrapped confidence intervals, δ_{Cliff}= Cliff's delta.

Table A4

Means, standard deviations, and correlations with confidence intervals GAD-7 Items

Variable	M	SD	1	2	3	4	5	6
1. #1	0.34	0.57						
2. #2	0.27	0.56	.60**					
			[.57, .62]					
3. #3	0.40	0.67	.56**	.72**				
			[.53, .59]	[.70, .74]				
4. #4	0.39	0.64	.57**	.58**	.62**			
			[.55, .60]	[.55, .61]	[.60, .65]			
5. #5	0.18	0.49	.52**	.53**	.47**	.57**		
			[.49, .54]	[.50, .56]	[.44, .50]	[.54, .59]		
6. #6	0.30	0.59	.52**	.54**	.56**	.58**	.54**	
			[.49, .55]	[.52, .57]	[.54, .59]	[.55, .60]	[.51, .57]	
#7	0.29	0.59	.58**	.61**	.65**	.55**	.55**	.57**
			[.55, .60]	[.59, .64]	[.62, .67]	[.52, .57]	[.52, .58]	[.55, .60]

N*ote.* Note. M and SD are used to represent mean and standard deviation, respectively. Values in square brackets indicate the 95% confidence interval. The confidence interval is a plausible range of population correlations that could have caused the sample correlation (Cumming, 2014). * indicates $p < .05$. ** indicates $p < .01$.

Table A5

Scale correlations: GAD-7, PHQ-9, BSI-18

Variable	M	SD	1	2	3	4	5
1. GAD-7	2.18	3.28					
2. PHQ-9	2.69	3.88	.85**				
			[.84, .86]				
3. BSI GSI	4.35	7.62	.76**	.79**			
			[.74, .78]	[.78, .81]			
4. BSI Somatization	1.21	2.53	.58**	.64**	.87**		
			[.55, .61]	[.62, .67]	[.86, .88]		
5. BSI Anxiety	1.25	2.54	.73**	.70**	.91**	.73**	
			[.71, .74]	[.68, .72]	[.91, .92]	[.71, 75]	
6. BSI Depression	1.89	3.38	.73**	.77**	.92**	.66**	.76**
			[.71, .75]	[.75, .79]	[.91, 92]	[.64, 68]	[.74, 77]

Note. GAD-7 = Generalized Anxiety scale; PHQ-9 = Patient Health Questionnaire; BSI GSI = Brief Symptom Inventory Global Severity Index; BSI Somatization = Brief Symptom Inventory Somatization Subscale; BSI Anxiety = Brief Symptom Inventory Anxiety Subscale; BSI Depression = Brief Symptom Inventory Depression Subscale; * indicates p < .05., ** indicates p < .001.

Table A6. GAD-7 *Item Characteristics*

Item	M	SD	Skew	Kurt	P	r_{IT}	α_if_deleted	λ
#1	0.34	0.57	1.67	3.05	11.46	0.69	0.89	0.84
#2	0.27	0.56	2.36	6.12	8.96	0.76	0.88	0.91
#3	0.40	0.67	1.75	2.83	13.34	0.75	0.88	0.90
#4	0.39	0.64	1.68	2.72	12.97	0.72	0.89	0.85
#5	0.18	0.49	3.14	11.00	6.10	0.65	0.89	0.84
#6	0.30	0.59	2.12	4.61	10.06	0.69	0.89	0.82
#7	0.29	0.59	2.26	5.19	9.62	0.73	0.89	0.87

Note: M = mean; *SD* = standard deviation; Skew = skewness; Kurt = kurtosis; *P* = percentage of participants endorsing the item; r_{IT} = item-total correlation; α _if_deleted = Cronbach's alpha if the item is deleted; λ = standardized factor loading.

Table A7

GAD-7 item-level associations with PHQ-9 and BSI-18 subscales

Item	PHQ-9		BSI-18 GSI		BSI-18 SOMA		BSI-18 ANX		BSI-18 DEPR	
	MIC	ϱ_s	MIC	ϱ_s	MIC	ϱ_s	MIC	ϱ_s	MIC	ϱ_s
#1	0.320	0.624	0.266	0.572	0.130	0.426	0.255	0.587	0.211	0.533
#2	0.328	0.609	0.247	0.534	0.128	0.409	0.177	0.474	0.235	0.547
#3	0.319	0.627	0.247	0.554	0.114	0.395	0.156	0.457	0.249	0.578
#4	0.319	0.633	0.237	0.545	0.108	0.384	0.209	0.535	0.194	0.511
#5	0.206	0.482	0.181	0.443	0.096	0.362	0.185	0.482	0.139	0.410
#6	0.272	0.574	0.204	0.494	0.108	0.386	0.160	0.459	0.177	0.486
#7	0.270	0.561	0.234	0.519	0.126	0.412	0.175	0.468	0.215	0.530

Note. GAD-7 = Generalized Anxiety Disorder-7; MIC = Maximum Information Coefficient; ϱ_s= Spearman's rank correlation coefficient; PHQ-9 = Patient Health Questionnaire-9; BSI-GSI = Brief Symptom Inventory – Global Severity Index; BSI-SOMA = Brief Symptom Inventory – Somatization Subscale; BSI-ANX = Brief Symptom Inventory – Anxiety Subscale; BSI-DEPR = Brief Symptom Inventory – Depression Subscale.

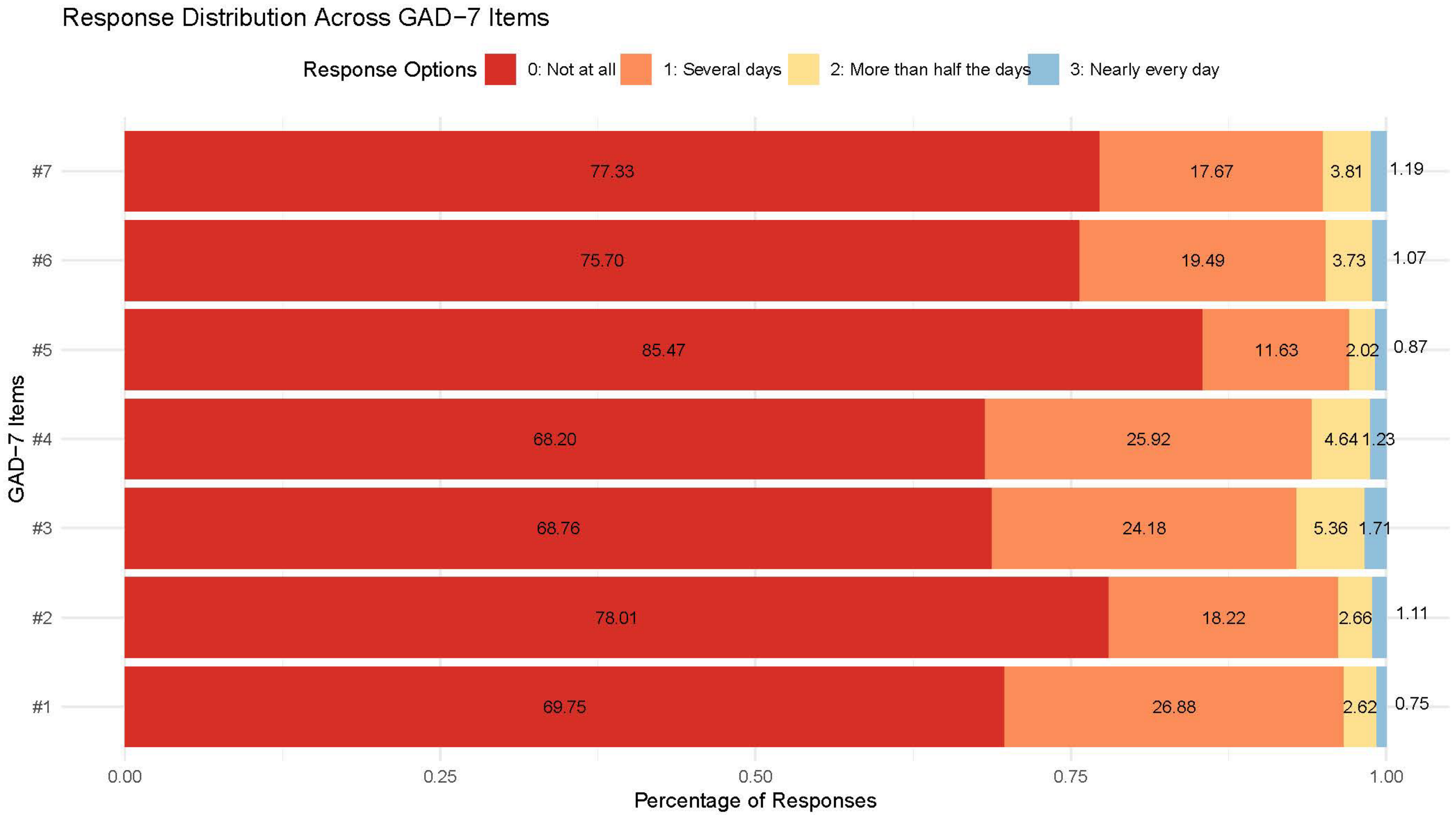

Figure A3. The figure displays the percentage distribution of responses across the seven GAD-7 items (#1–#7). Each horizontal bar is segmented by response options—0 ("Not at all"), 1 ("Several days"), 2 ("More than half the days"), and 3 ("Nearly every day")—with each segment's percentage (rounded to two decimals) representing its relative frequency.

Table A8

Smoothed population based norms of the GAD-7 (total sample)

GAD-7	Total	age 16-24	age 25-34	age 35-44	age 45-54	age 55-64	age 65-74	age 75+
0	47.8	53.4	55	51.9	43.3	42.8	47.6	41.7
1	60.8	61.1	68.5	63.3	60	55.4	61.6	54.8
2	69.8	65.8	75	71.8	69.7	66.5	71.2	67.8
3	76.3	70.9	79.6	78.3	76.1	75.3	77.8	74.3
4	81.7	77.2	83.8	83.3	81.4	81.3	82.6	78.6
5	86.2	83.4	88.1	87.1	86.1	85.3	86.6	83.8
6	89.8	88.7	91.9	89.8	90.7	88.2	90.4	87.9
7	92.5	92.4	95	91.7	94.2	90.9	93.6	90.1
8	94.5	94.5	97	93	96.2	93.3	95.4	91.8
9	95.9	95.5	98.1	93.9	97.3	95.3	96.2	93.5
10	96.9	96.2	98.7	94.7	98	96.5	96.7	95.7
11	97.6	96.9	99.1	95.5	98.5	97.1	97.5	97.9
12	98.1	97.6	99.4	96.2	98.9	97.5	98.2	99.4
13	98.5	98.3	99.7	96.9	99.2	97.7	98.8	> 99.9
14	98.8	99	> 99.9	97.5	99.5	98	99.2	> 99.9
15	99.1	99.6	> 99.9	98	99.8	98.2	99.5	> 99.9
16	99.3	> 99.9	> 99.9	98.5	> 99.9	98.5	99.7	> 99.9
17	99.5	> 99.9	> 99.9	98.9	> 99.9	98.8	> 99.9	> 99.9
18	99.7	> 99.9	> 99.9	99.2	> 99.9	99	> 99.9	> 99.9
19	99.8	> 99.9	> 99.9	99.5	> 99.9	99.3	> 99.9	> 99.9
20	> 99.9	> 99.9	> 99.9	99.8	> 99.9	99.6	> 99.9	> 99.9
21	> 99.9	> 99.9	> 99.9	> 99.9	> 99.9	99.9	> 99.9	> 99.9

Table A9

Population based norms of the GAD-7 (male subsample)

GAD-7	Total	age 16-24	age 25-34	age 35-44	age 45-54	age 55-64	age 65-74	age 75+
0	51.6	59.8	63.7	55.7	44.1	46.7	48.4	45.9
1	63.5	68.6	76.3	65.5	60.0	57.4	61.6	56.1
2	73.5	70.6	81.1	76.4	68.7	70.5	75.8	69.4
3	79.0	77.5	84.2	82.8	74.4	79.1	78.9	73.5
4	83.4	82.4	87.9	88.5	79.5	82.0	82.6	79.6
5	87.4	93.1	90.0	90.2	85.1	84.8	86.8	83.7
6	90.7	96.1	93.2	93.1	87.7	88.1	91.6	86.7
7	94.0	97.1	96.3	94.3	93.8	92.6	94.2	89.8
8	95.4	97.1	98.4	95.4	95.9	93.4	95.8	90.8
9	96.2	99.0	98.4	96.6	96.9	94.7	95.8	91.8
10	97.1	99.0	98.4	97.1	98.5	95.5	96.8	93.9
11	98.1	99.0	99.5	97.7	99.0	96.3	97.4	99.0
12	98.2	99.0	99.5	97.7	99.0	96.3	97.9	> 99.9
13	98.7	> 99.9	> 99.9	98.3	99.0	96.7	98.9	> 99.9
14	99.0	> 99.9	> 99.9	98.3	99.5	97.1	99.5	> 99.9
16	99.2	> 99.9	> 99.9	98.3	> 99.9	97.5	99.5	> 99.9
17	99.4	> 99.9	> 99.9	98.9	> 99.9	98.0	> 99.9	> 99.9
18	99.6	> 99.9	> 99.9	98.9	> 99.9	98.8	> 99.9	> 99.9
19	99.7	> 99.9	> 99.9	99.4	> 99.9	98.8	> 99.9	> 99.9
20	99.7	> 99.9	> 99.9	> 99.9	> 99.9	98.8	> 99.9	> 99.9
21	> 99.9	> 99.9	> 99.9	> 99.9	> 99.9	> 99.9	> 99.9	> 99.9

Table A10

Smoothed population based norms of the GAD-7 (male subsample)

GAD-7	Total	age 16-24	age 25-34	age 35-44	age 45-54	age 55-64	age 65-74	age 75+
0	51.5	59.9	63.7	55.6	44.2	46.3	48.2	45.9
1	64.1	67.7	76.3	66.1	59.8	58.9	63.1	57.4
2	72.7	71.7	81	75.7	68.6	69.4	73.1	67.2
3	78.9	76.8	84.5	83.2	74.6	77.5	79.6	74.3
4	83.8	83.8	87.5	88	79.6	82.8	83.9	79.5
5	87.8	91.2	90.2	90.9	84.4	86.3	87.3	83.4
6	90.9	96	93.2	92.8	89.1	88.8	90.6	86.4
7	93.3	97.6	96.2	94.3	93	91	93.5	88.9
8	95.1	98.1	98	95.5	95.6	93	95.4	91
9	96.4	98.4	98.6	96.5	97.1	94.5	96.4	93.1
10	97.3	98.8	98.9	97.1	98.1	95.6	97.1	95.3
11	97.9	99.1	99.2	97.5	98.7	96.2	97.7	97.6
12	98.3	99.4	99.6	97.8	99.1	96.6	98.2	> 99.9
13	98.6	99.8	> 99.9	98.1	99.4	96.9	98.6	> 99.9
14	98.9	> 99.9	> 99.9	98.3	99.7	97.2	99	> 99.9
16	99.3	> 99.9	> 99.9	98.7	> 99.9	97.8	99.8	> 99.9
17	99.4	> 99.9	> 99.9	98.9	> 99.9	98.1	> 99.9	> 99.9
18	99.6	> 99.9	> 99.9	99.1	> 99.9	98.5	> 99.9	> 99.9
19	99.7	> 99.9	> 99.9	99.4	> 99.9	98.8	> 99.9	> 99.9
20	99.9	> 99.9	> 99.9	99.8	> 99.9	99.2	> 99.9	> 99.9
21	> 99.9	> 99.9	> 99.9	100	> 99.9	99.8	> 99.9	> 99.9

Table A11

Population based norms of the GAD-7 (female subsample)

GAD-7	Total	age 16-24	age 25-34	age 35-44	age 45-54	age 55-64	age 65-74	age 75+
0	44.3	48.0	45.4	48.9	42.6	39.5	47.0	38.8
1	57.9	56.0	60.3	61.8	60.2	52.3	60.5	52.5
2	67.0	61.6	68.4	68.0	70.4	63.0	69.0	67.6
3	74.6	66.4	73.6	75.1	77.8	75.3	76.0	74.1
4	79.5	71.2	81.0	79.1	82.9	79.4	81.5	77.7
5	84.6	78.4	84.5	84.4	87.0	84.4	87.0	84.2
6	88.4	82.4	90.2	87.6	93.1	85.6	89.5	88.5
7	92.5	90.4	94.3	90.2	95.4	92.2	94.0	89.9
8	94.1	92.0	96.6	90.7	96.3	94.7	95.0	92.8
9	95.2	93.6	97.7	91.6	97.2	95.9	95.5	95.0
10	96.4	95.2	97.7	93.3	98.1	97.1	96.5	96.4
11	97.1	96.0	98.9	93.8	98.1	97.9	98.0	97.1
12	97.8	96.0	99.4	95.1	98.6	97.9	98.5	99.3
13	98.4	98.4	99.4	96.0	99.1	98.4	98.5	> 99.9
14	99.0	99.2	> 99.9	96.4	> 99.9	99.2	99.0	> 99.9
15	99.5	> 99.9	> 99.9	98.2	> 99.9	99.2	99.5	> 99.9
16	99.6	> 99.9	> 99.9	98.7	> 99.9	99.2	> 99.9	> 99.9
17	99.7	> 99.9	> 99.9	99.1	> 99.9	99.2	> 99.9	> 99.9
18	99.8	> 99.9	> 99.9	99.6	> 99.9	99.6	> 99.9	> 99.9
19	99.9	> 99.9	> 99.9	99.6	> 99.9	> 99.9	> 99.9	> 99.9
21	> 99.9	> 99.9	> 99.9	> 99.9	> 99.9	> 99.9	> 99.9	> 99.9

Table A12

Smoothed population based norms of the GAD-7 (female subsample)

GAD-7	Total	age 16-24	age 25-34	age 35-44	age 45-54	age 55-64	age 65-74	age 75+
0	44.3	48.3	45.5	49.1	42.7	39.5	47.1	38.5
1	57.9	55.5	60.1	61.1	60	51.9	60.2	53.1
2	67.2	61.4	68.2	68.9	70.7	64.2	69.1	67.3
3	74	66.5	74.4	74.5	77.5	73.9	76	73.8
4	79.8	71.7	79.9	79.6	82.7	79.9	81.8	78.2
5	84.8	77.8	85.4	84.1	88	83.8	86.5	84
6	88.8	84	90.3	87.5	92.4	87.4	90.3	88.3
7	91.8	88.8	93.8	89.8	95.1	91	93.1	90.4
8	94	91.8	96.2	91.1	96.4	94.1	94.8	92.5
9	95.4	93.7	97.5	92	97.3	96.1	96	94.9
10	96.5	95	98.3	92.9	97.8	97.2	96.9	96.5
11	97.2	96.1	98.8	93.9	98.3	97.8	97.6	97.4
12	97.8	97	99.3	94.9	98.8	98.2	98.2	98.8
13	98.4	98	99.7	96	99.3	98.5	98.7	> 99.9
14	98.9	99	> 99.9	97	99.8	98.8	99.1	> 99.9
15	99.3	> 99.9	> 99.9	97.9	> 99.9	99.1	99.6	> 99.9
16	99.6	> 99.9	> 99.9	98.5	> 99.9	99.3	> 99.9	> 99.9
17	99.8	> 99.9	> 99.9	99	> 99.9	99.5	> 99.9	> 99.9
18	99.8	> 99.9	> 99.9	99.4	> 99.9	99.7	> 99.9	> 99.9
19	99.9	> 99.9	> 99.9	99.7	> 99.9	99.9	> 99.9	> 99.9
21	> 99.9	> 99.9	> 99.9	> 99.9	> 99.9	> 99.9	> 99.9	> 99.9

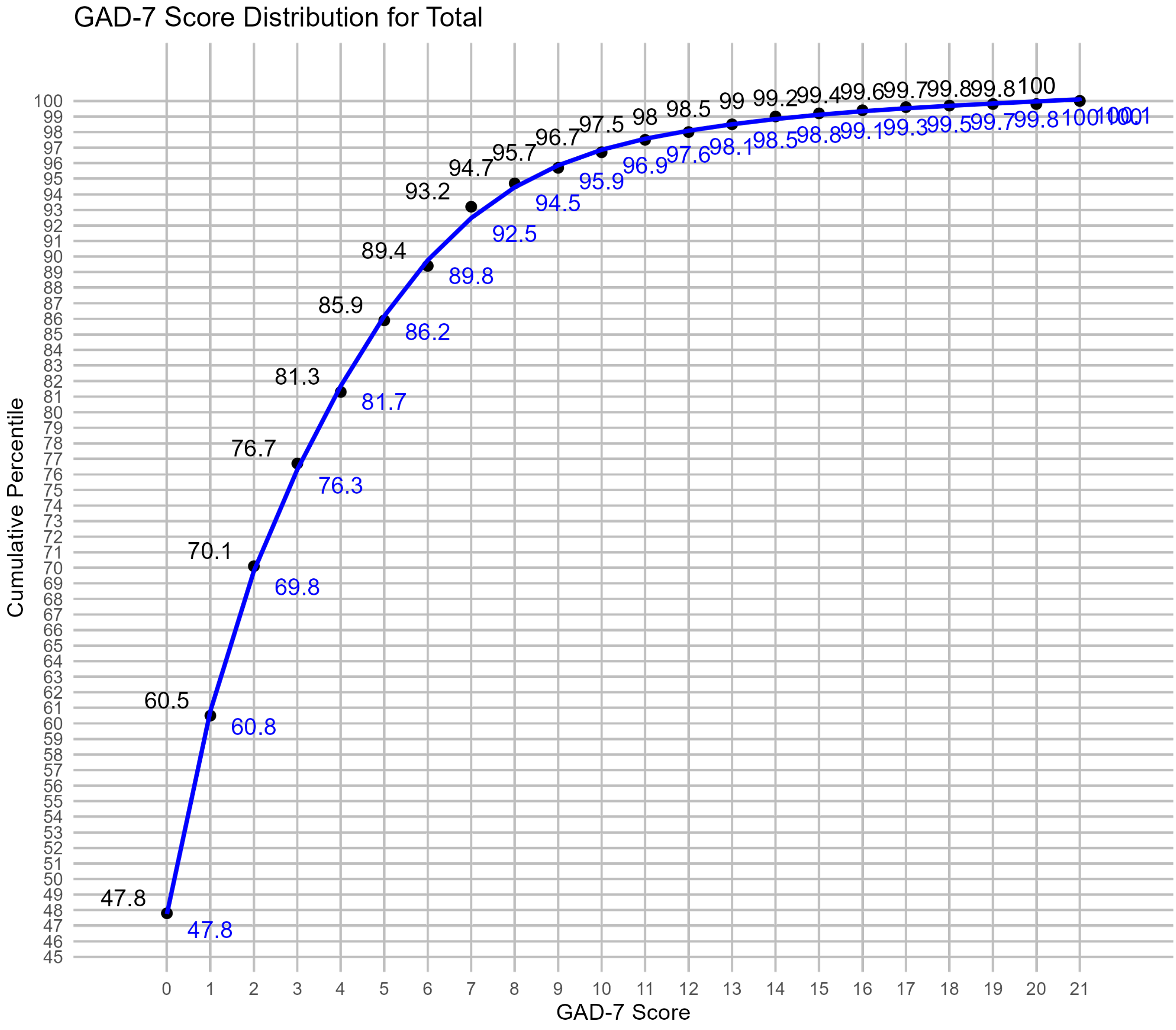

Figure A4: Distribution of GAD-7 Scores for the Total Sample. The blue dots show the observed cumulative percentiles the blue line is SCAM-smoothed.

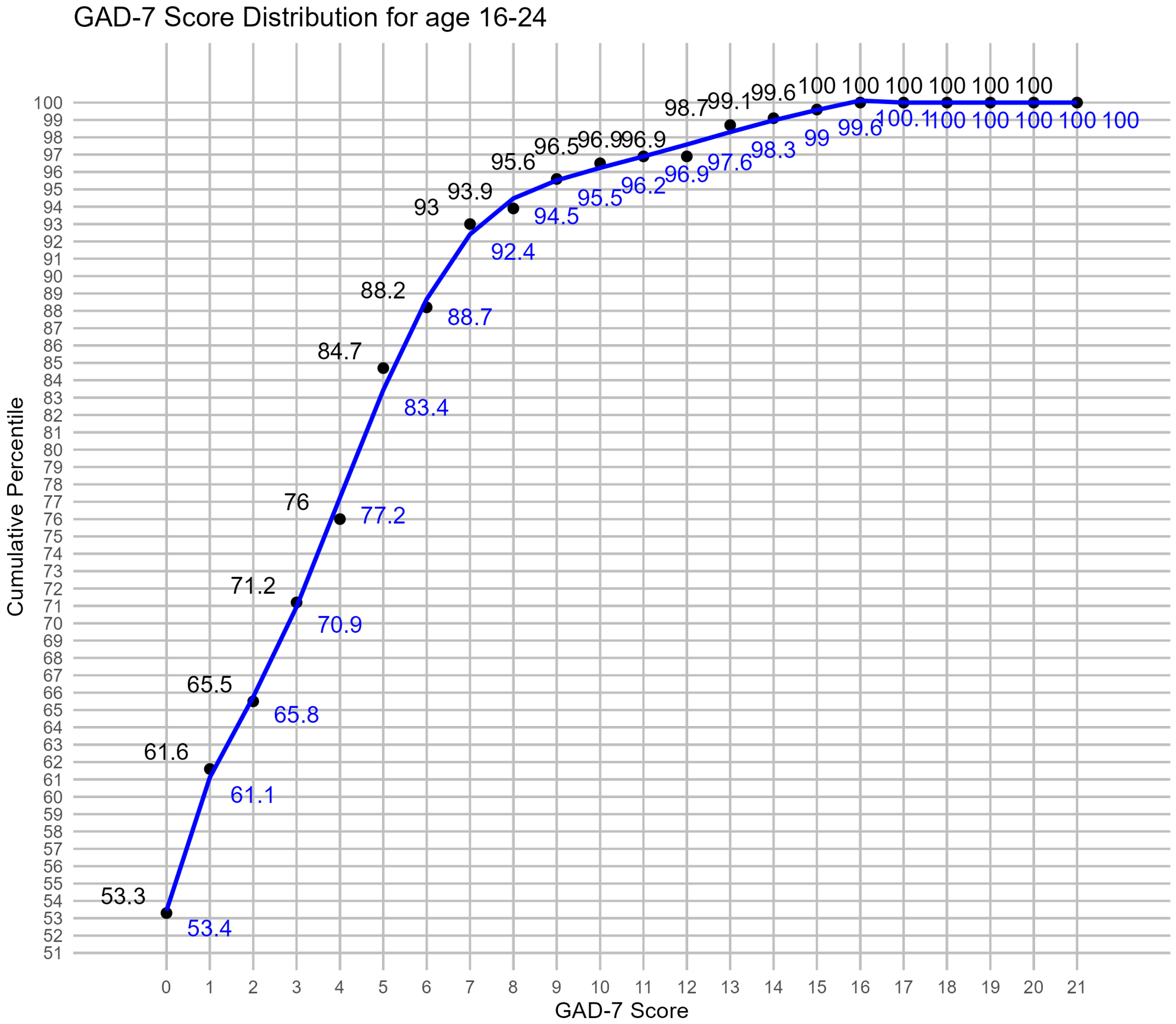

Figure A5: Distribution of GAD-7 Scores for Age Group 16–24. The blue dots show the observed cumulative percentiles the blue line is SCAM-smoothed.

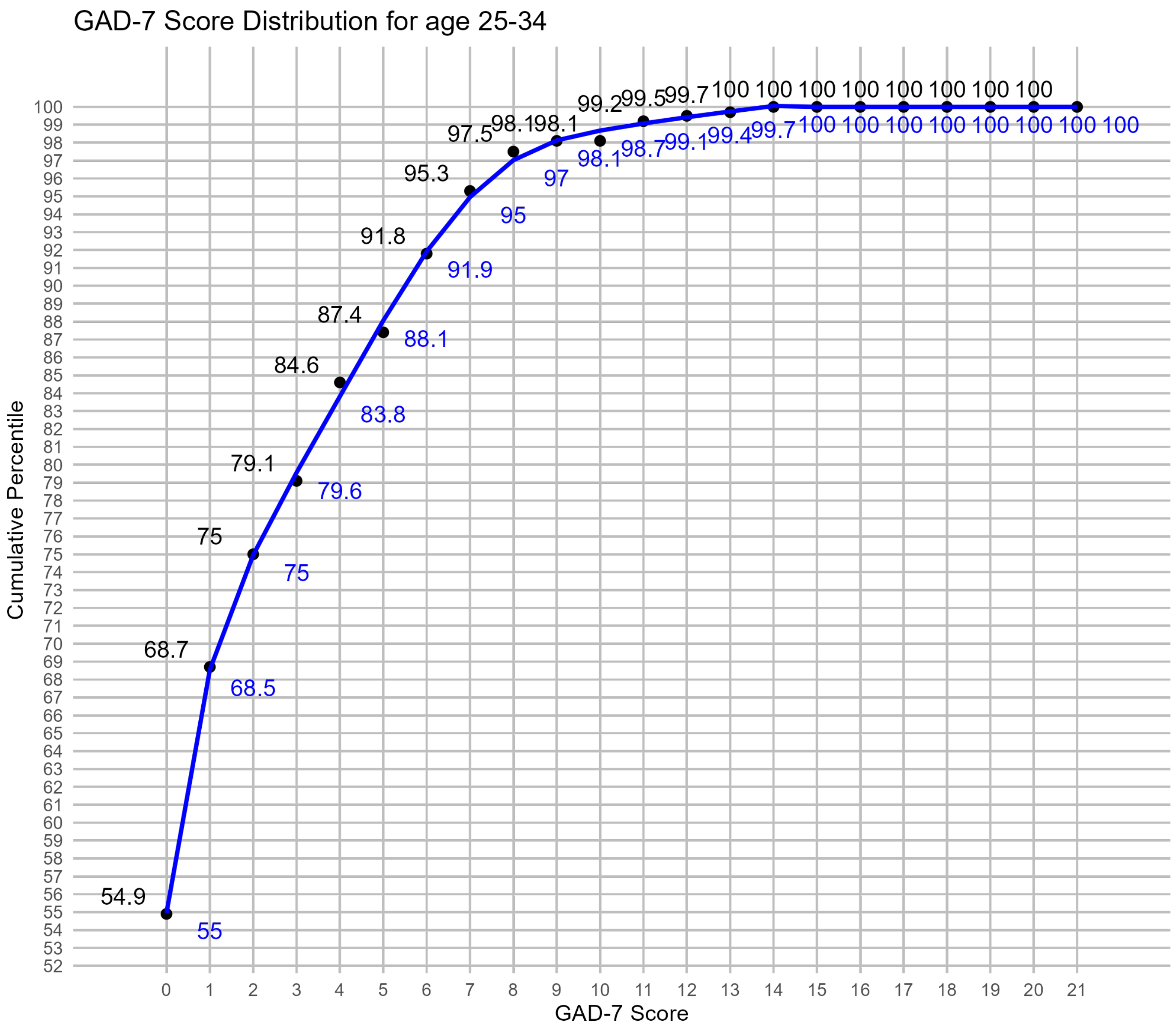

Figure A6: Distribution of GAD-7 Scores for Age Group 25–34. The blue dots show the observed cumulative percentiles the blue line is SCAM-smoothed.

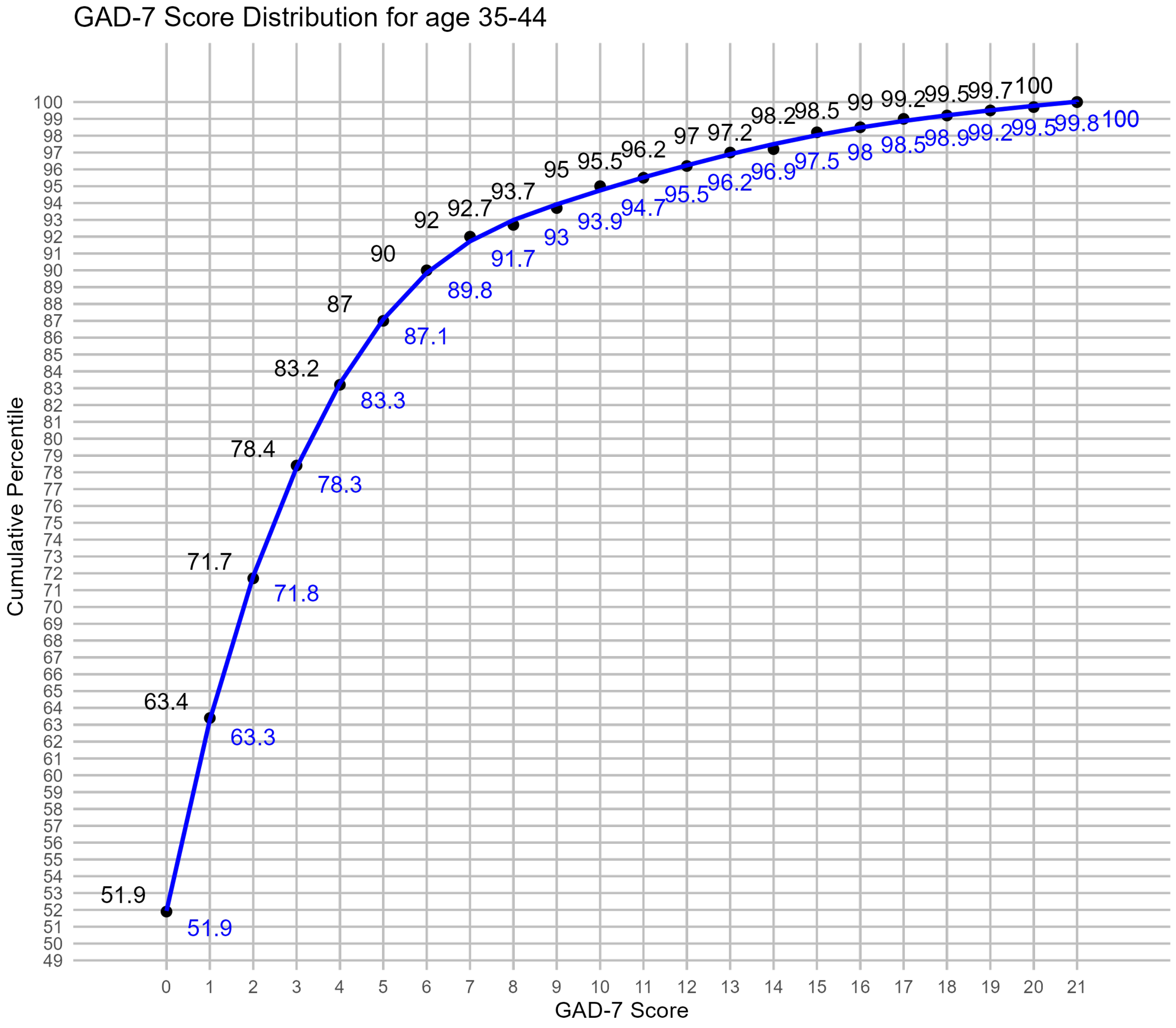

Figure A7: Distribution of GAD-7 Scores for Age Group 35–44. The blue dots show the observed cumulative percentiles the blue line is SCAM-smoothed.

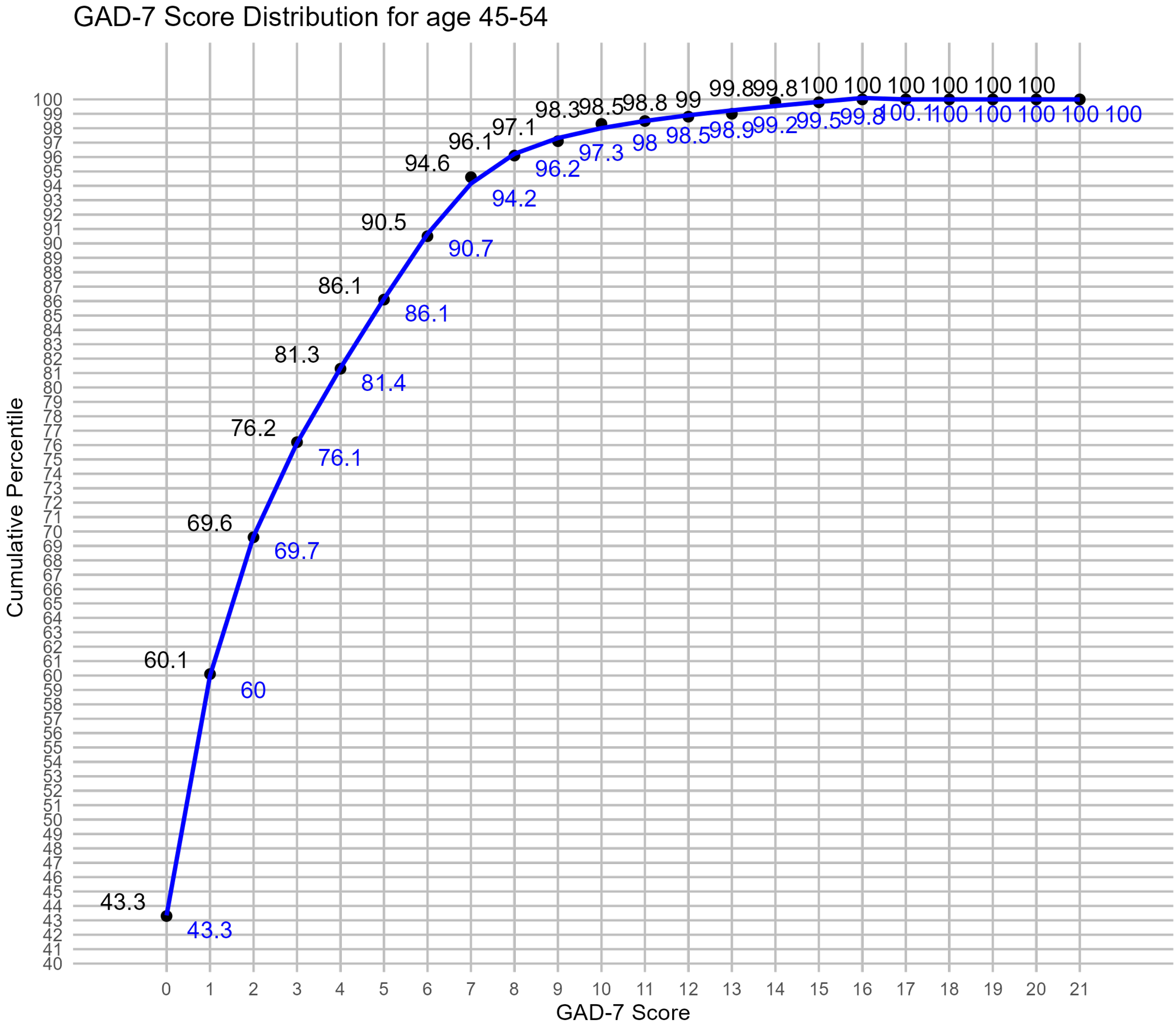

Figure A8: Distribution of GAD-7 Scores for Age Group 45–54. The blue dots show the observed cumulative percentiles the blue line is SCAM-smoothed.

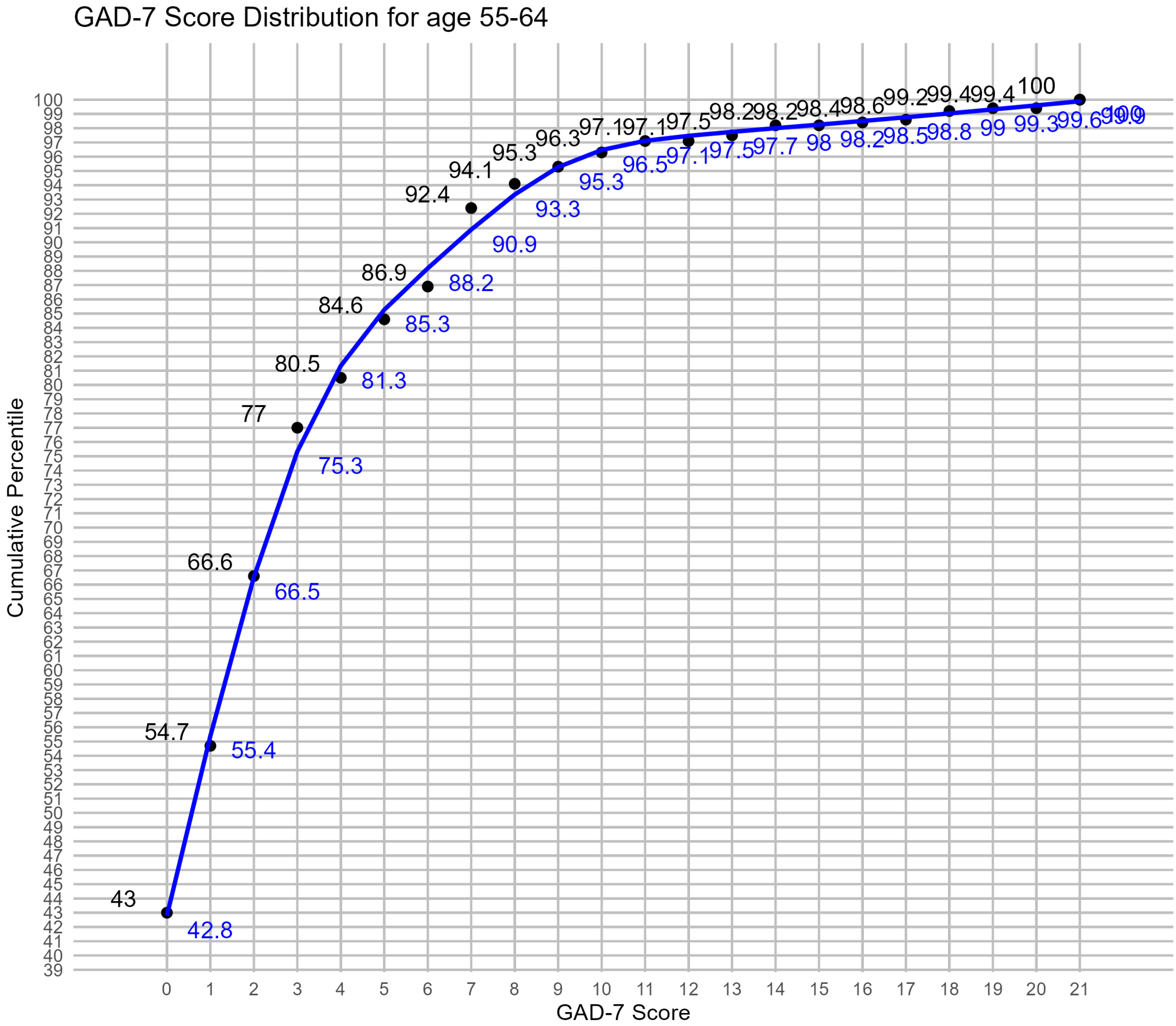

Figure A9: Distribution of GAD-7 Scores for Age Group 55–64. The blue dots show the observed cumulative percentiles the blue line is SCAM-smoothed.

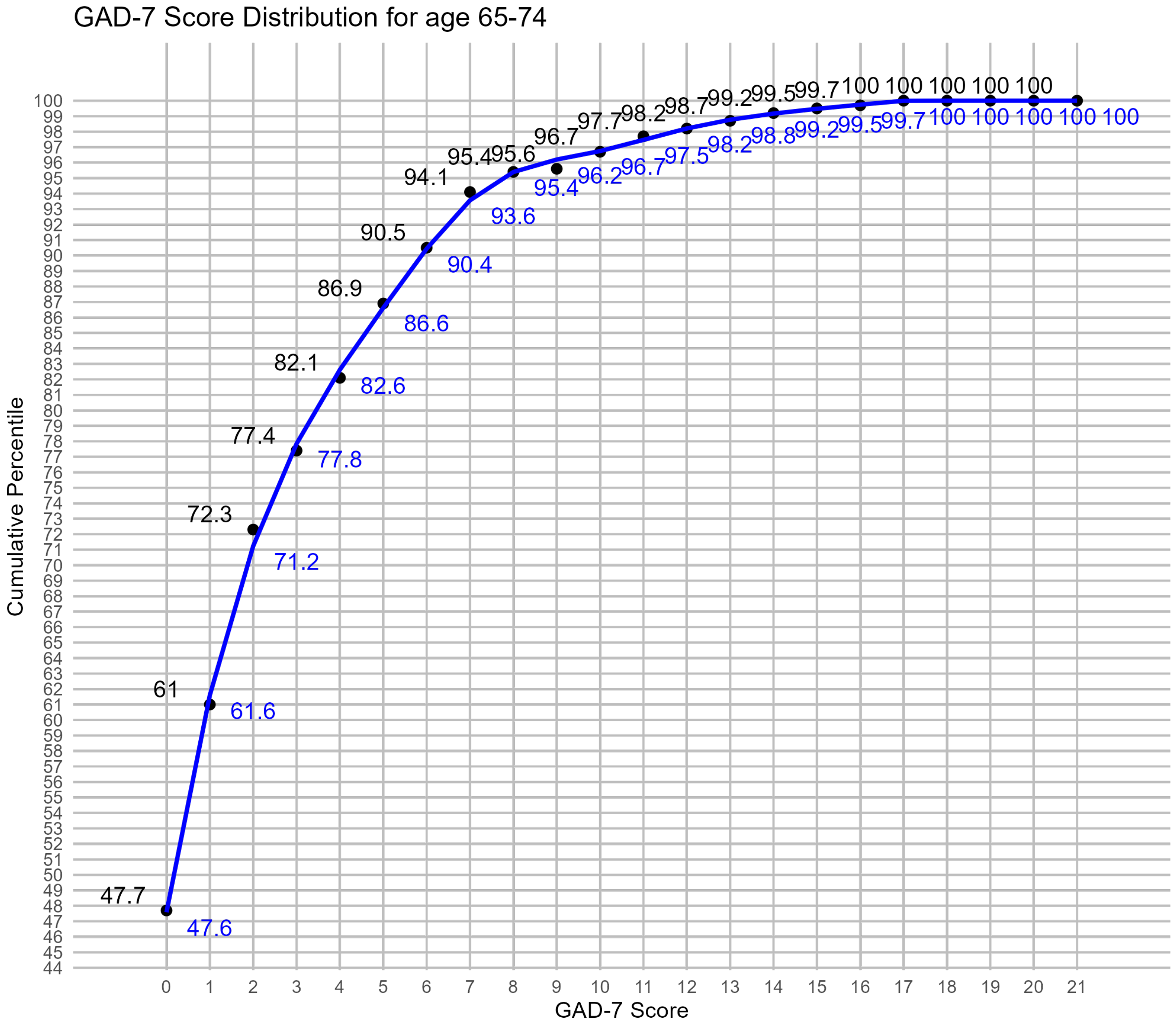

Figure A10: Distribution of GAD-7 Scores for Age Group 65–74. The blue dots show the observed cumulative percentiles the blue line is SCAM-smoothed.

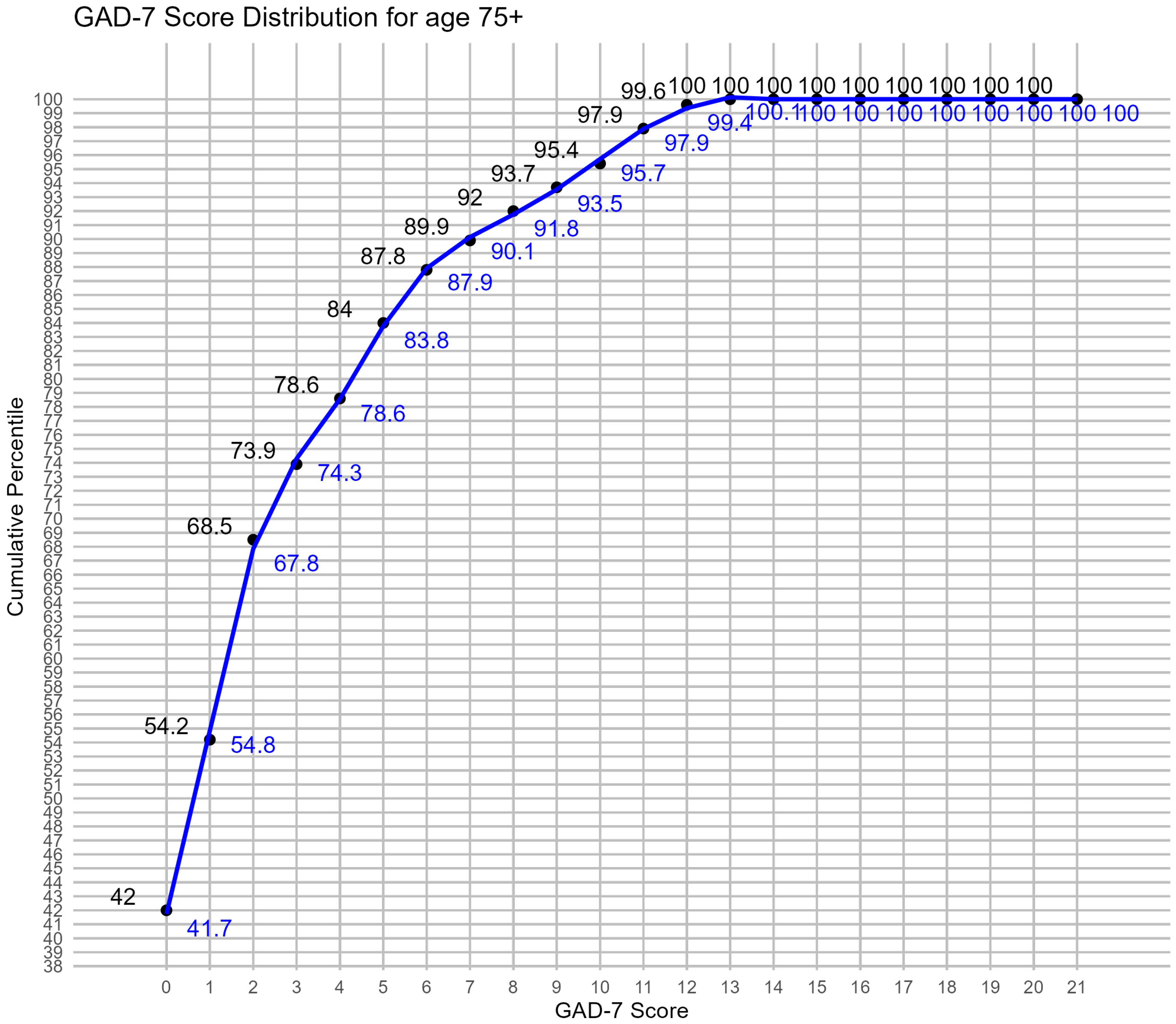

Figure A11: Distribution of GAD-7 Scores for Age Group 75+. The blue dots show the observed cumulative percentiles the blue line is SCAM-smoothed.

B Sensitivity analysis

To evaluate the robustness of the results, a sensitivity analysis was conducted by excluding respondents with inconsistent response patterns. Outliers were identified based on G+ scores, a measure of Guttman errors, which occur when respondents endorse harder items but not easier ones, violating the expected hierarchical order of items (van der Ark, 2012). The G+ scores were calculated using the `check.errors()` function from the **`mokken`** package in R.

Outliers were defined using Tukey's method, with scores exceeding the upper fence (Q3+3×IQR) flagged as discordant (Tukey, 1977). A total of 87 outliers (3.5% of the sample) were excluded, as inconsistent response patterns can bias psychometric results and distort conclusions. Analyses were repeated without these cases to assess the sensitivity of the findings.

References

- Tukey, J. W. (1977). *Exploratory Data Analysis*. Addison-Wesley.
- van der Ark, L. A. (2012). New developments in Mokken scale analysis in R. *Journal of Statistical Software, 48*(5), 1–27.

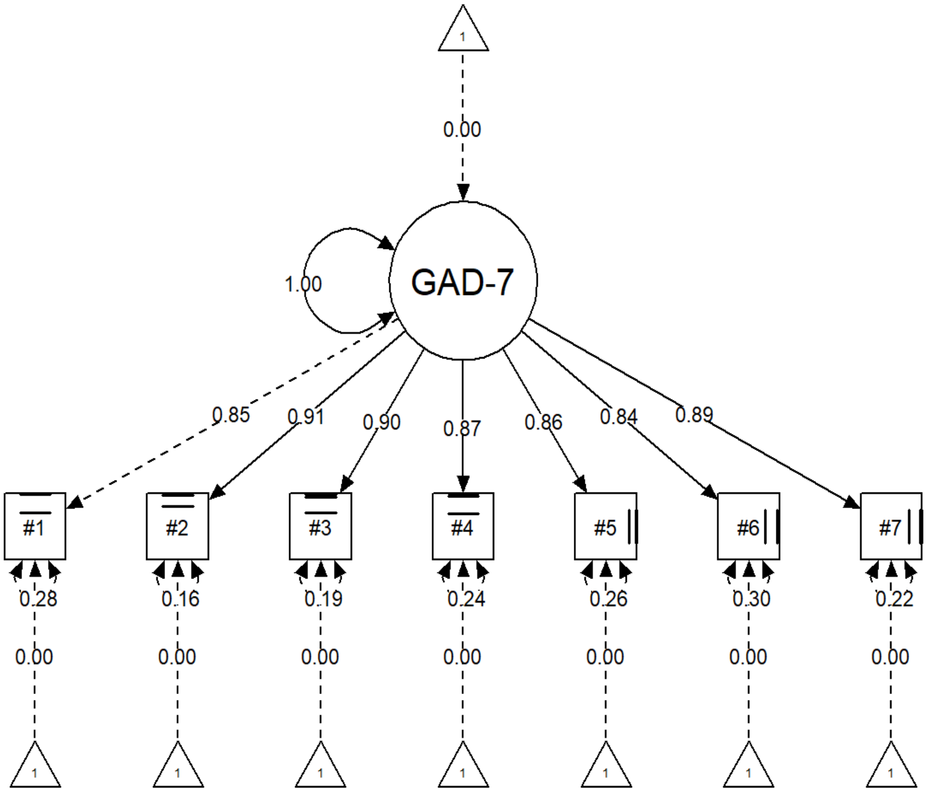

Figure B1. One factor CFA model of the GAD-7.

Table B2

Scale correlations: GAD-7, PHQ-9, BSI-18

Variable	M	SD	1	2	3	4	5
1. GAD-7	1.94	3.02					
2. PHQ-9	2.47	3.62	.85**				
			[.84, .86]				
3. BSI GSI	3.96	7.14	.75**	.79**			
			[.74, .77]	[.77, .80]			
4. BSI Somatization	1.12	2.39	.58**	.65**	.87**		
			[.56, .61]	[.62, .67]	[.86, .88]		
5. BSI Anxiety	1.13	2.36	.72**	.70**	.91**	.72**	
			[.70, .74]	[.68, .72]	[.91, .92]	[.70, .74]	
6. BSI Depression	1.70	3.17	.72**	.76**	.92**	.66**	.77**
			[.70, .74]	[.74, .78]	[.91, .93]	[.64, .69]	[.75, .78]

Note. GAD-7 = Generalized Anxiety scale; PHQ-9 = Patient Health Questionnaire; BSI GSI = Brief Symptom Inventory Global Severity Index; BSI Somatization = Brief Symptom Inventory Somatization Subscale; BSI Anxiety = Brief Symptom Inventory Anxiety Subscale; BSI Depression = Brief Symptom Inventory Depression Subscale; * indicates $p < .05$., ** indicates $p < .001$.

Table B3

Percentage of participants per severity level based on Spitzer et al, 2006 GAD-7 cut-offs

GAD-7 severity	Total, %	Male, %	Female, %
minimal	83.96	85.94	82.22
mild	12.99	11.28	14.57
moderate	2.18	1.74	2.58
severe	0.86	1.04	0.63

Table 3

Population based norms (cumulative percentiles) of the GAD-7 scores (total sample)

GAD-7	Total	age 16-24	age 25-34	age 35-44	age 45-54	age 55-64	age 65-74	age 75+
0	49.5	56.5	56.5	53.5	44.7	44.6	48.9	44.2
1	62.7	65.3	70.6	65.4	62.1	56.7	62.6	57.1
2	72.5	69.4	77.1	73.9	71.9	68.8	74.2	72.1
3	79.3	75	81.4	80.9	78.6	79.4	79.5	77.9
4	84	79.6	86.7	85.8	83.9	82.8	84.2	82.7
5	88.3	88.4	89.3	89.4	88.7	86.4	88.7	87.6
6	91.3	91.2	93.2	91.5	92.5	88.5	92.4	90.3
7	94.7	94.9	96.3	93	96.2	93.6	95.8	92
8	96.1	95.8	98.3	93.8	97.7	95.1	97.1	93.8
9	97	97.2	98.9	94.6	98.5	96.4	97.4	95.6
10	97.7	98.1	98.9	95.9	99	97.2	97.9	97.3
11	98.3	98.1	99.4	96.4	99.2	97.7	98.7	99.1
12	98.6	98.1	99.7	97.2	99.2	97.7	99.2	> 99.9
13	98.9	99.1	99.7	97.7	99.5	98.1	99.2	> 99.9
14	99.1	99.5	> 99.9	97.9	99.7	98.3	99.2	> 99.9
15	99.3	99.5	> 99.9	98.4	99.7	98.3	99.5	> 99.9
16	99.5	> 99.9	> 99.9	98.7	> 99.9	98.5	99.7	> 99.9
17	99.6	> 99.9	> 99.9	99	> 99.9	98.7	> 99.9	> 99.9
18	99.7	> 99.9	> 99.9	99.2	> 99.9	99.2	> 99.9	> 99.9
19	99.8	> 99.9	> 99.9	99.5	> 99.9	99.4	> 99.9	> 99.9
20	99.8	> 99.9	> 99.9	99.7	> 99.9	99.4	> 99.9	> 99.9
21	> 99.9	> 99.9	> 99.9	> 99.9	> 99.9	> 99.9	> 99.9	> 99.9

Table B4. *Results of measurement invariance analyses*

	χ^2	df	CFI	*Δ*CFI	RMSEA	*Δ*RMSEA	Measurement invariance
Gender (male, female)							
Configural invariance	78.377	28	0.999	-	0.062	-	-
Threshold invariance [1]	85.759	35	0.999	0	0.055	-0.007	✓
Metric invariance [2]	86.513	41	0.999	0	0.049	-0.006	✓
Scalar invariance [3]	92.949	47	0.999	0	0.045	-0.004	✓
Full invariance [4]	99.592	54	0.999	0	0.037	-0.008	✓
Age (<= 51, >51)							
Configural invariance	90.944	28	0.999	NA	0.067	-	-
Threshold invariance [1]	97.221	35	0.999	0	0.059	-0.009	✓
Metric invariance [2]	99.093	41	0.999	0	0.052	-0.006	✓
Scalar invariance [3]	117.76	47	0.998	0	0.052	0	✓
Full invariance [4]	127.231	54	0.998	0	0.044	-0.008	✓
Age x Gender							
Configural invariance	94.387	56	0.999	-	0.062	-	-
Threshold invariance [1]	104.968	74	0.999	0	0.051	-0.011	✓
Metric invariance [2]	110.158	92	1	0	0.043	-0.009	✓
Scalar invariance [3]	142.759	110	0.999	0	0.044	0.001	✓
Full invariance [4]	168.629	131	0.999	0	0.036	-0.008	✓

Note. All fit statistics are robust; CFI = Comparative Fit Index; CFI = CFI-differences for the different measurement invariance levels; RMSEA = Root Mean Square Error of Approximation; RMSEA = RMSEA- differences for the different measurement invariance levels; = *Δ*CFI < -.010 complemented by RMSEA ≥ .015 indicates a violation of measurement invariance; marks measurement invariance for the respective level; [1] equivalency of thresholds; [2] equivalency of thresholds + factor loadings; [3] equivalency of thresholds + factor loadings + equivalency of constants; [4] equivalency of thresholds + factor loadings + equivalency of constants + unique-factor variances.

Table B6. *Population based norms of the GAD-7 (male subsample)*

GAD-7	Total	age 16-24	age 25-34	age 35-44	age 45-54	age 55-64	age 65-74	age 75+
0	53.5	62.9	66.1	56.4	45.7	48.9	49.7	47.9
1	65.8	72.2	79.2	66.3	62.2	60.1	63.2	58.5
2	76	74.2	84.2	77.3	71.3	73.4	77.8	72.3
3	81.6	80.4	87.4	83.7	77.1	82	81.1	76.6
4	85.9	84.5	91.3	89.5	82.4	84.5	84.9	83
5	89.6	95.9	92.9	91.3	87.8	86.7	88.6	86.2
6	92.3	97.9	95.1	93	89.9	89.7	93.5	88.3
7	95.2	99	97.8	94.2	95.2	93.6	96.2	90.4
8	96.5	99	99.5	95.3	97.3	94.4	97.8	91.5
9	97.2	> 99.9	99.5	96.5	97.9	95.7	97.8	92.6
10	97.9	> 99.9	99.5	97.1	98.9	96.6	98.4	94.7
11	98.6	> 99.9	> 99.9	97.7	99.5	96.6	98.9	98.9
12	98.8	> 99.9	> 99.9	97.7	99.5	96.6	99.5	> 99.9
13	99	> 99.9	> 99.9	98.3	99.5	97	99.5	> 99.9
16	99.1	> 99.9	> 99.9	98.3	> 99.9	97.4	99.5	> 99.9
17	99.4	> 99.9	> 99.9	98.8	> 99.9	97.9	> 99.9	> 99.9
18	99.6	> 99.9	> 99.9	98.8	> 99.9	98.7	> 99.9	> 99.9
19	99.7	> 99.9	> 99.9	99.4	> 99.9	98.7	> 99.9	> 99.9
20	99.7	> 99.9	> 99.9	> 99.9	> 99.9	98.7	> 99.9	> 99.9
21	> 99.9	> 99.9	> 99.9	> 99.9	> 99.9	> 99.9	> 99.9	> 99.9

Table B7. *Population based norms of the GAD-7 (female subsample)*

GAD-7	Total	age 16-24	age 25-34	age 35-44	age 45-54	age 55-64	age 65-74	age 75+
0	45.8	51.3	46.2	51.2	43.8	40.3	48.2	41.2
1	59.9	59.8	61.4	64.7	61.9	53.4	62.1	55.7
2	69.4	65.8	69.6	71.2	72.4	64.3	70.8	71.8
3	77.2	70.9	74.9	78.6	80	76.9	77.9	78.6
4	82.2	76.1	81.9	82.8	85.2	81.1	83.6	82.4
5	87.2	82.9	85.4	87.9	89.5	86.1	88.7	88.5
6	90.5	86.3	91.2	90.2	94.8	87.4	91.3	91.6
7	94.2	92.3	94.7	92.1	97.1	93.7	95.4	93.1
8	95.7	94	97.1	92.6	98.1	95.8	96.4	95.4
9	96.8	95.7	98.2	93	99	97.1	96.9	97.7
10	97.7	97.4	98.2	94.9	99	97.9	97.4	99.2
11	98.1	97.4	98.8	95.3	99	98.7	98.5	99.2
12	98.6	97.4	99.4	96.7	99	98.7	99	> 99.9
13	99	99.1	99.4	97.2	99.5	99.2	99	> 99.9
14	99.4	> 99.9	> 99.9	97.7	> 99.9	99.6	99	> 99.9
15	99.6	> 99.9	> 99.9	98.6	> 99.9	99.6	99.5	> 99.9
16	99.8	> 99.9	> 99.9	99.1	> 99.9	99.6	> 99.9	> 99.9
18	99.8	> 99.9	> 99.9	99.5	> 99.9	99.6	> 99.9	> 99.9
19	99.9	> 99.9	> 99.9	99.5	> 99.9	> 99.9	> 99.9	> 99.9
21	> 99.9	> 99.9	> 99.9	> 99.9	> 99.9	> 99.9	> 99.9	> 99.9
